# Supplementary material for: Unveiling a large fraction of hidden organosulfates in ambient organic aerosol
Source: Nat Commun. 2025 May 1;16:4098. doi: 10.1038/s41467-025-59420-y (PMC12046013; doi:10.1038/s41467-025-59420-y)
Supplement: Supplementary file 1 — Supplementary Information [file 41467_2025_59420_MOESM1_ESM.pdf]

Supplementary Information

Unveiling a large fraction of hidden organosulfates in ambient organic aerosol

Jialiang Ma[1*], Natalie Reininger[1,2], Cunliang Zhao[3], Damian Döbler[4], Julian Rüdiger[4], Yanting Qiu[5], Florian Ungeheuer[1], Mario Simon[1], Luca D'Angelo[1], Anna Breuninger[1], Julia David[1], Yanxin Bai[6], Yushan Li[6], Ying Xue[6], Lili Li[6], Yuchen Wang[6], Stefanie Hildmann[7], Thorsten Hoffmann[7], Bangjun Liu[3], Hongya Niu[3], Zhijun Wu[5], and Alexander L. Vogel[1*]

[1]Institute for Atmospheric and Environmental Sciences, Goethe-University Frankfurt, Frankfurt am Main, Germany

[2]Institute for Ecology, Evolution and Diversity, Goethe University Frankfurt, Frankfurt am Main, Germany

[3]School of Earth Science and Engineering, Hebei University of Engineering, Handan, China

[4]Air Monitoring Network, German Environment Agency, Langen, Germany

[5]State Key Joint Laboratory of Environmental Simulation and Pollution Control, Peking University, Beijing, China

[6]College of Environmental Science and Engineering, Hunan University, Changsha, China

[7]Institute of Inorganic and Analytical Chemistry, Johannes Gutenberg-University, Mainz, Germany

[*]e-mail: ma@iau.uni-frankfurt.de; vogel@iau.uni-frankfurt.de

Document Contains:

51 Pages

18 Figures

9 Tables

Table of contents

Figure S1. Evaluation of the SPE isolation recovery and reproducibility 3

Figure S2. Molecular fingerprints of the enriched OS fractions 4

Figure S3. Comparison of the CAD and HRMS response 5

Figure S4. Calibration surface of CAD 6

Figure S5. Matrix suppression test 7

Figure S6. CAD baseline fitting 8

Figure S7. CAD peak fitting 9

Figure S8. Comparison of the RT and MS/MS fragmentation patterns of octyl sulfate 10

Figure S9. Complete CAD chromatogram 11

Figures S10-14. Comparison of HRMS and CAD chromatograms 12

Figure S15. Correlation of UHPLC-CAD and HRMS chromatograms 17

Figure S16. Backward trajectories 18

Figure S17. SPE-recovery of isoprene-derived OSs 19

Figure S18. Schematic of the filter preparation 20

Tables S1-6 Quantitive numbers of all OSs in measured samples 21

Table S7. Summary of the OS fraction in OM quantified by various techniques 36

Tables S8-9. Compound Discoverer workflow 37

Supplementary References 51

Figure S1. Evaluation of the SPE isolation recovery and reproducibility

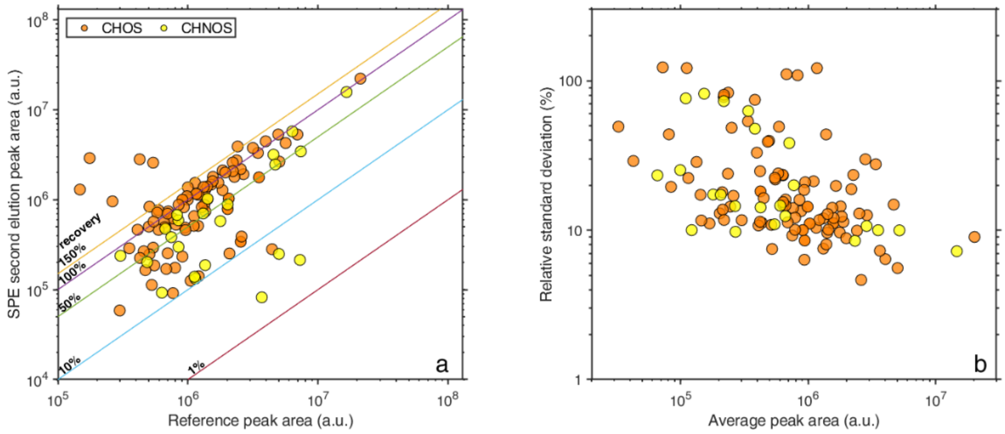

Fig. S1: Evaluation of the SPE isolation recovery (a) and reproducibility (b) for individual compounds using a $PM_{2.5}$ filter extract from Handan (18.10.2018, daytime). We measure the all of the native extract, first SPE elution, second SPE elution, and flow through of Handan 1 (18.10.2018, daytime) in triplicated to evaluate the relative standard deviation of the SPE method.

Figure S2. Molecular fingerprints of the enriched OS fractions

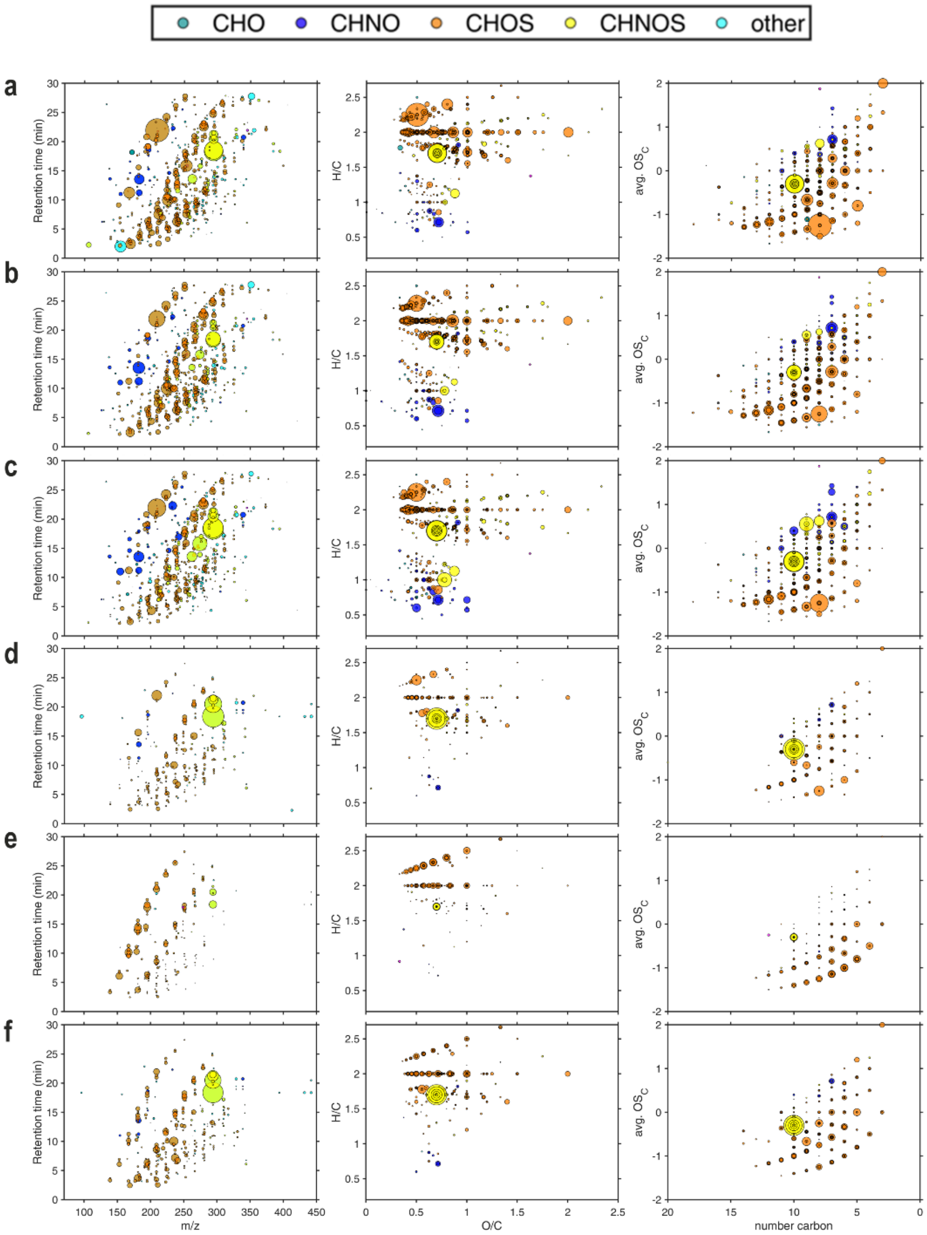

Fig. S2: Molecular fingerprints (*m/z*-RT plot, Van Krevelen diagram, and Kroll diagram) of the enriched OS fractions. (a) Handan 1: 18.10.2018, daytime; (b) Handan 2: 21.10.2018, daytime; (c) Handan 3: 21.10.2018, night-time; (d) TO 1: 11.12.2021, daytime; (e) TO 2: 14.01.2022, night-time; (f) TO 3: 28.02.2022, night-time.

Figure S3. Comparison of the CAD and HRMS response

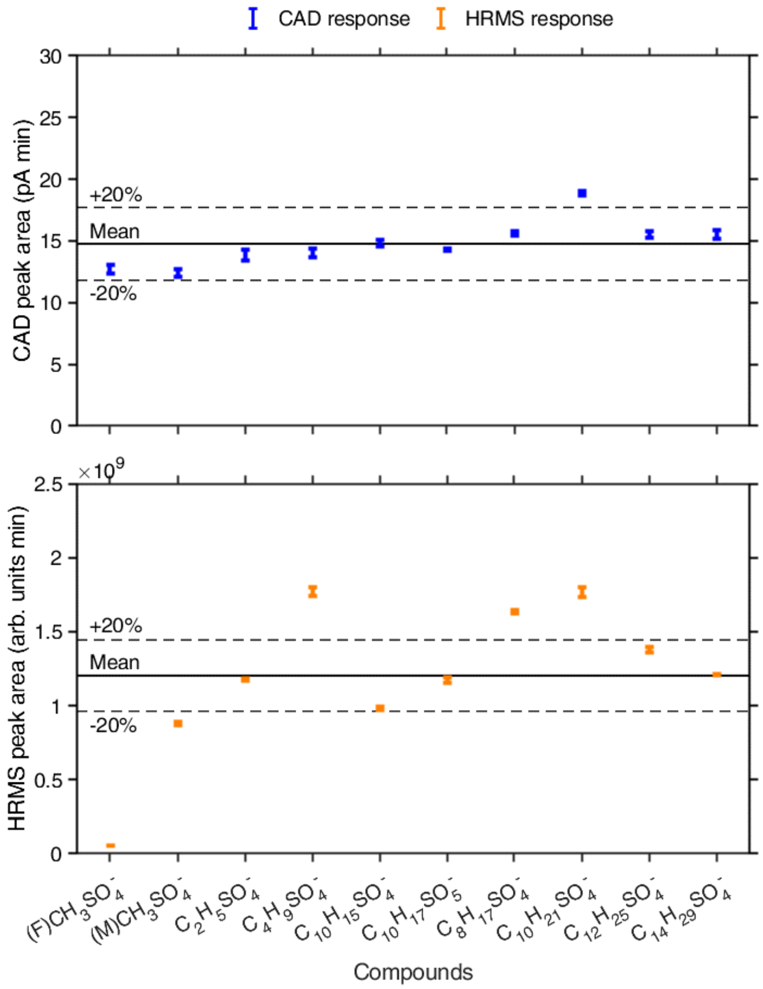

Fig. S3: Comparison of the CAD (blue, upper figure) and HRMS (orange, lower figure) response (mean ± SD) of ten OS standards at an isocratic gradient. The standards were prepared in one batch as an OS-mix. The mix contained: (1) Formaldehyde sodium bisulfite (CH_3SO_4Na), (2) Methyl sulfate sodium salt (CH_3SO_4Na), (3) Sodium ethyl sulfate ($C_2H_5SO_4Na$), (4) Butyl sulfate sodium salt ($C_4H_{10}SO_4Na$), (5) Camphor-10-sulfonic acid ($C_{10}H_{16}SO_4$), (6) 2-hydroxy-α-pinene OS ($C_{10}H_{18}SO_5$), (7) Sodium octyl sulfate ($C_8H_{17}SO_4Na$), (8) Sodium n-decyl sulfate ($C_{10}H_{21}SO_4Na$), (9) Sodium dodecyl sulfate ($C_{12}H_{25}SO_4Na$), and (10) Tetradecyl sulfate sodium salt ($C_{14}H_{29}SO_4Na$). Each OS standard had a concentration of 2 ng/µL. To test the stability of the instruments, we measured each standard concentration five times. The HPLC flowrate was set to 0.4 mL/min, with 83.5% being directed into the CAD and 16.5% being directed into the MS. The line (solid) is the average response of these compounds with a band (dash) of ± 20%.

Figure S4. Calibration surface of CAD

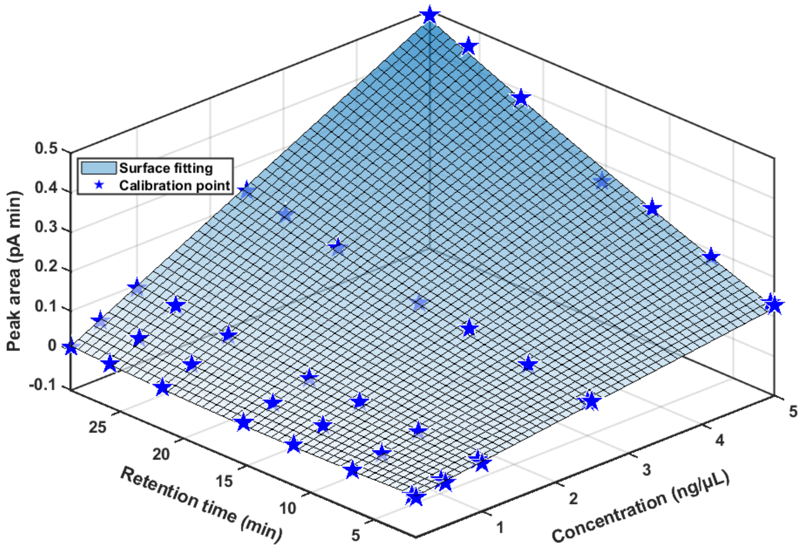

Fig. S4: Calibration surface fitted by OS standards. CAD measurement of eight different standards at five altered concentrations. The 40 data points were fitted by using a 2nd order polynomial function ($z = p00 + p10x + p01y + p11xy + p02y^2$ ($z = peak\ area, y = RT\ (min), x = concentration\ (ng/\mu L),\ p00 = 0.005127,\ p10 = 0.01947,\ p01 = -0.001816, p11 = 0.002637, p02 = 0.000059$)) to get the calibration surface. Standards from low to high RT: Methyl sulfate sodium salt (RT: 1.70), Sodium ethyl sulfate (RT: 2.04), Butyl sulfate (RT: 6.73), Camphor-10-sulfonic acid (RT: 11.38), 2-hydroxy-α-pinene OS (RT: 15.33), Sodium octyl sulfate (RT: 21.73), Sodium n-decyl sulfate (RT: 25.87), Sodium dodecyl sulfate (RT: 28.93).

Figure S5. Matrix suppression test

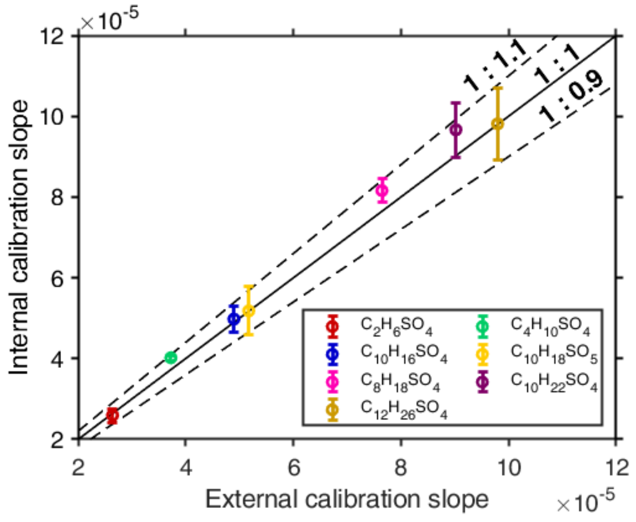

Fig. S5: Comparison of the internal and external calibration curve. The error bar indicates the standard deviation of the six samples.

Figure S6. CAD baseline fitting

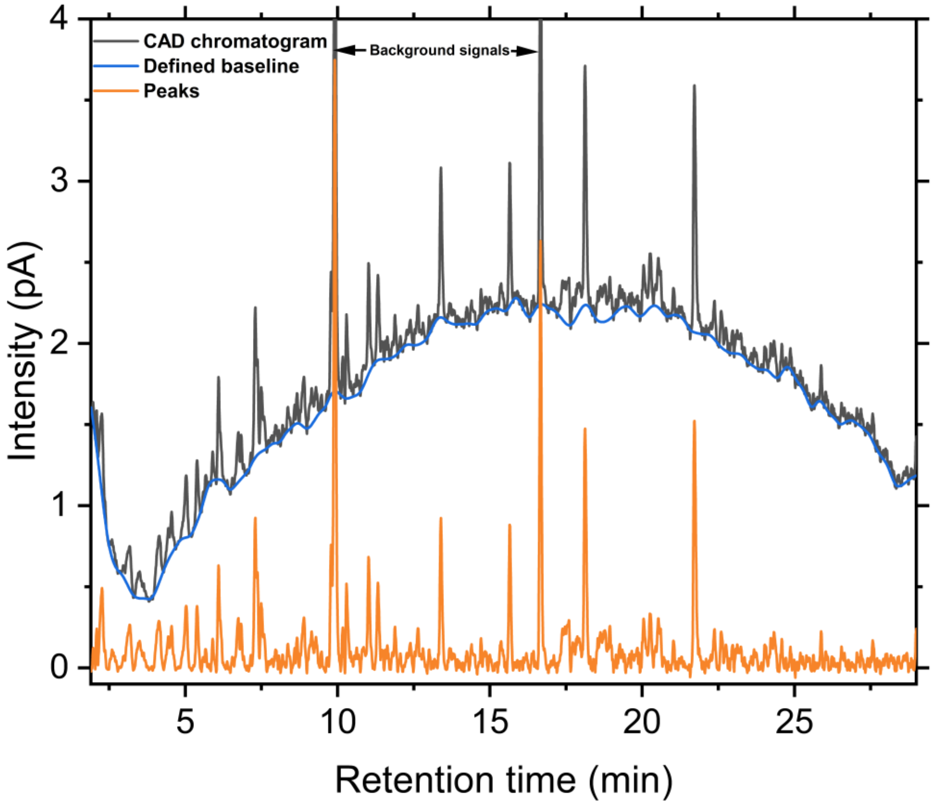

Fig. S6: Baseline fitting in the CAD chromatogram (Sample: Handan 18.10.2018, daytime). The blank-subtracted CAD chromatogram (grey) is fitted by a running baseline (blue). Subtraction of the baseline from the chromatogram results in a chromatogram solely representing the chromatographically-resolved peaks (orange).

Figure S7. CAD peak fitting

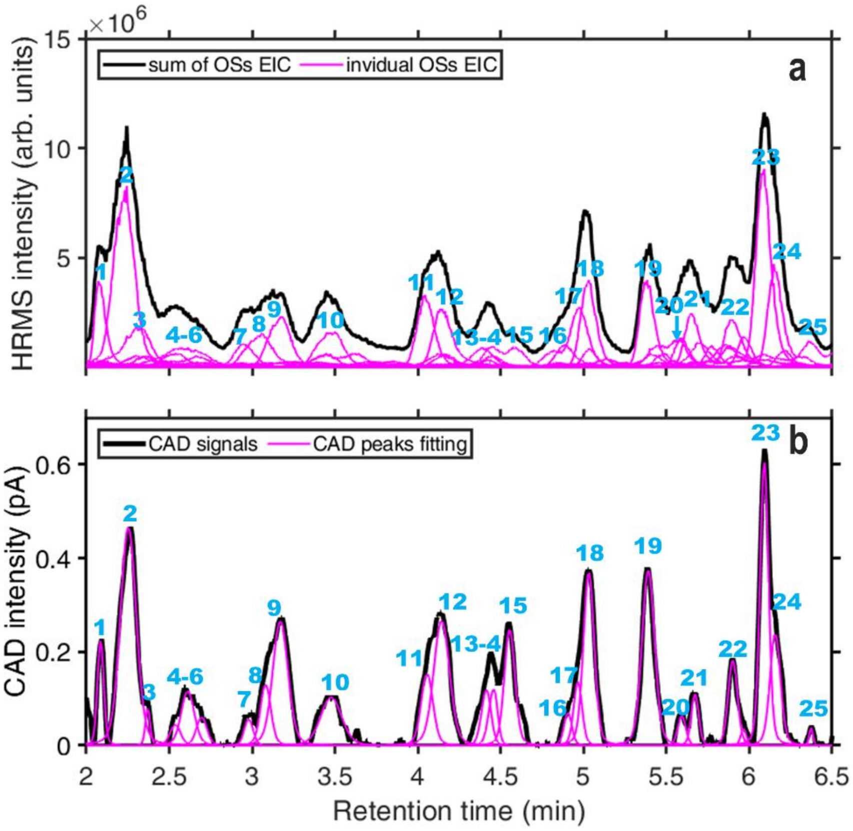

Fig. S7: CAD peak fitting by using the EIC of OSs as a reference (sample: Handan 18.10.2018, daytime). We used Fityk to fit the peaks and within RT range between 2-6.5 min as an example. (a) The indices are the notes for the reference peaks in HRMS. (b) The indices are the separated peak fittings of various compounds.

Figure S8. Comparison of the RT and MS/MS fragmentation patterns of octyl sulfate

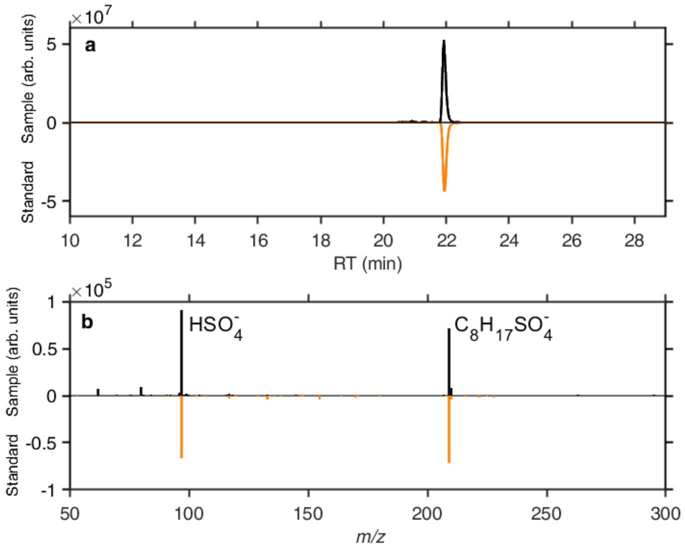

Fig. S8: Comparison of the (a) retention time (RT) and (b) MS/MS fragmentation patterns of octyl sulfate ($C_8H_{18}SO_4$) measured in an ambient sample (Handan, 18.10.2018 daytime, upward spectra, black) and their standards (downward spectra, orange).

Figure S9. Complete CAD chromatogram

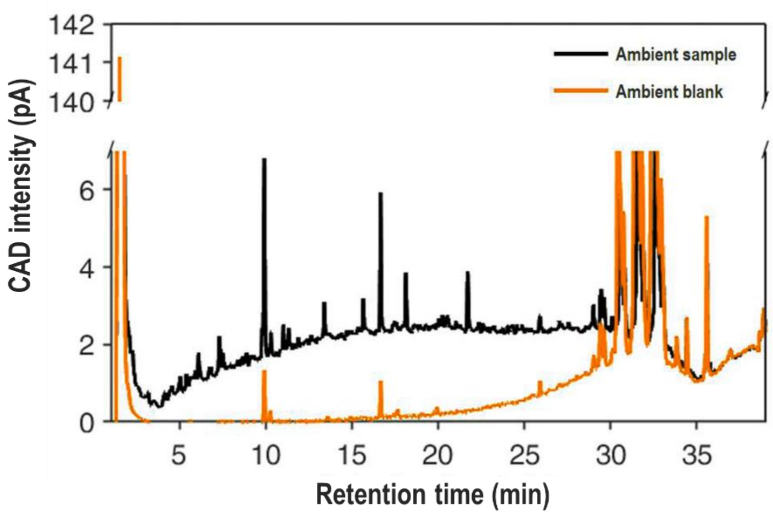

Fig. S9: Comparison of the UHPLC-CAD chromatograms (complete chromatograms) of the enriched OS fraction (second SPE elution) of the ambient filter sample (black line), and the enriched OS fraction (second SPE elution) of the according ambient filter blank (orange line).

Figures S10-14. Comparison of HRMS and CAD chromatograms

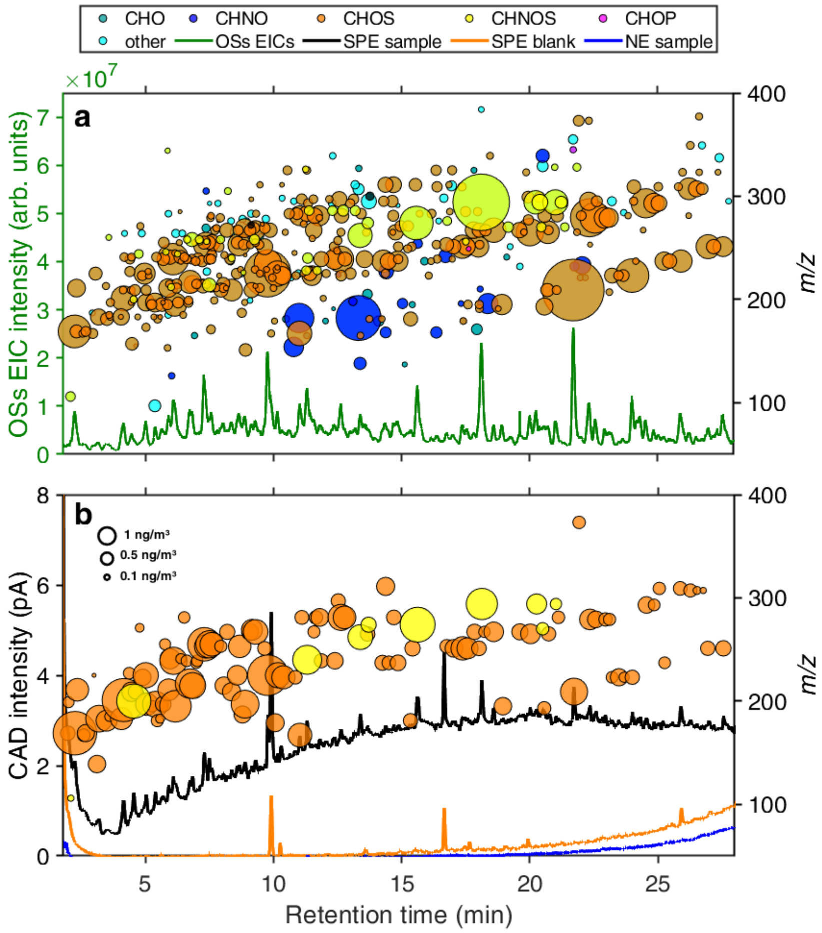

Fig. S10: Comparison of the chromatograms and fingerprints of the enriched OS fraction, measured by UHPLC-HRMS (a) and UHPLC-CAD (b), of a Handan filter (Handan 2, 21.10.2018 daytime). The x-axis, displaying the retention time (min), is zoomed in to 2.5-28 min of a total method duration of 40 min. There was no integration possible for OS CAD peaks outside of this time window. **(a)** UHPLC-HRMS data of the enriched OS fraction: RT-*m/z* plot (fingerprints, right y-axis) and the sum of the extracted ion chromatograms (EIC, green line, left y-axis) of all the OS fingerprints appearing in the RT-*m/z* plot. The circle size represents the OS intensity. **(b)** Comparison of the UHPLC-CAD chromatograms of the enriched OS fraction (second SPE elution) of the ambient filter sample (SPE sample, black line), the enriched OS fraction of the according ambient filter blank (SPE blank, orange line), and the native extraction (regular liquid extraction technique) of the same ambient filter sample (NE sample, blue line) with the molecular fingerprints of the quantifiable OSs of the SPE sample. The circle size represents the OS concentration, determined from the three-dimensional calibration (Fig. S4).

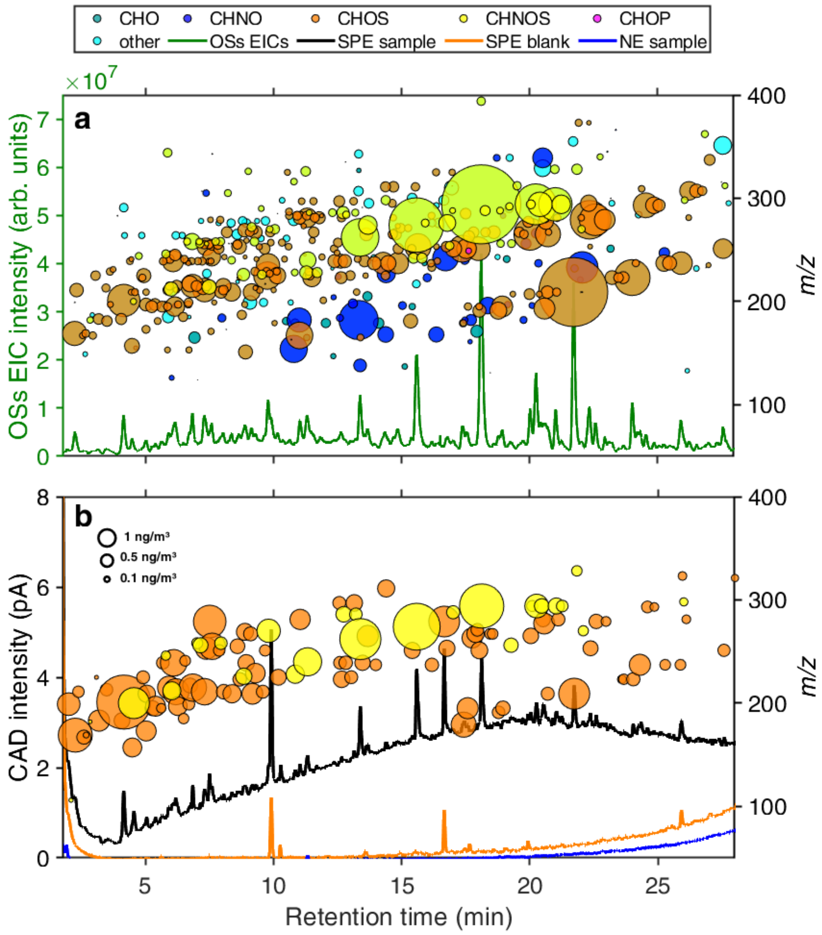

Fig. S11: Comparison of the chromatograms and fingerprints of the enriched OS fraction, measured by UHPLC-HRMS (a) and UHPLC-CAD (b), of a Handan filter (Handan 3, 21.10.2018 night-time). The x-axis, displaying the retention time (min), is zoomed in to 2.5-28 min of a total method duration of 40 min. There was no integration possible for OS CAD peaks outside of this time window. **(a)** UHPLC-HRMS data of the enriched OS fraction: RT-*m/z* plot (fingerprints, right y-axis) and the sum of the extracted ion chromatograms (EIC, green line, left y-axis) of all the OS fingerprints appearing in the RT-*m/z* plot. The circle size represents the OS intensity. **(b)** Comparison of the UHPLC-CAD chromatograms of the enriched OS fraction (second SPE elution) of the ambient filter sample (SPE sample, black line), the enriched OS fraction of the according ambient filter blank (SPE blank, orange line), and the native extraction (regular liquid extraction technique) of the same ambient filter sample (NE sample, blue line) with the molecular fingerprints of the quantifiable OSs of the SPE sample. The circle size represents the OS concentration, determined from the three-dimensional calibration (Fig. S4).

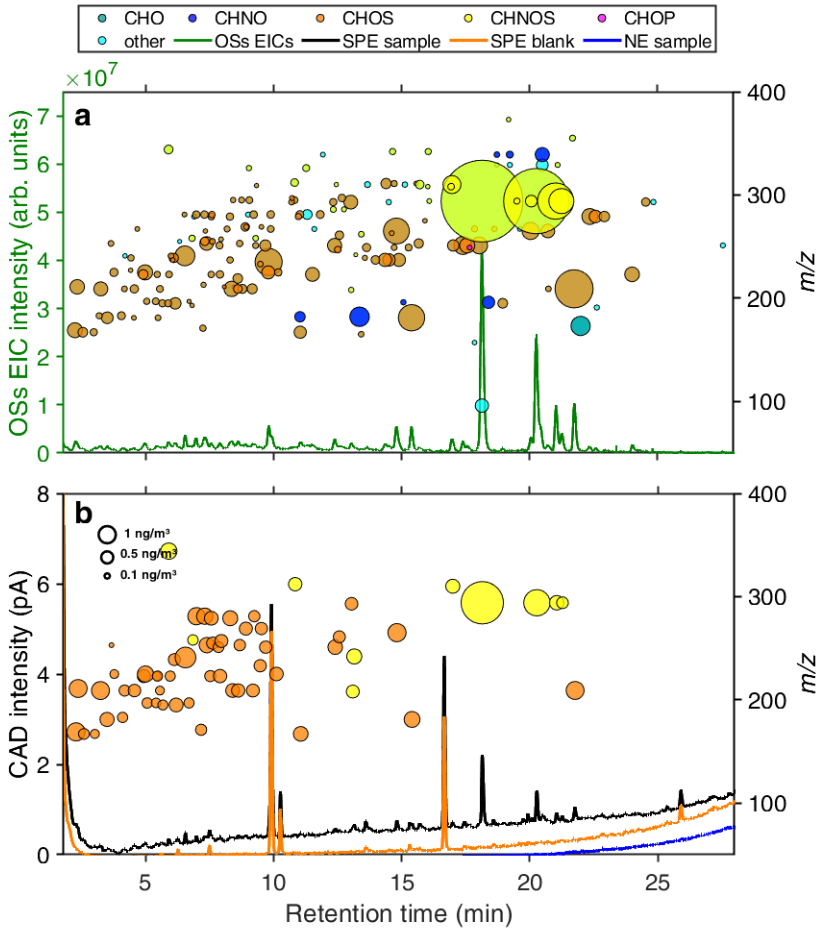

Fig. S12: Comparison of the chromatograms and fingerprints of the enriched OS fraction, measured by UHPLC-HRMS (a) and UHPLC-CAD (b), of a TO filter (TO 1, 11.12.2021 daytime). The x-axis, displaying the retention time (min), is zoomed in to 2.5-28 min of a total method duration of 40 min. There was no integration possible for OS CAD peaks outside of this time window. The complete CAD chromatogram is shown in Fig. S8. **(a)** UHPLC-HRMS data of the enriched OS fraction: RT-*m/z* plot (fingerprints, right y-axis) and the sum of the extracted ion chromatograms (EIC, green line, left y-axis) of all the OS fingerprints appearing in the RT-*m/z* plot. The circle size represents the OS intensity. **(b)** Comparison of the UHPLC-CAD chromatograms of the enriched OS fraction (second SPE elution) of the ambient filter sample (SPE sample, black line), the enriched OS fraction of the according ambient filter blank (SPE blank, orange line), and the native extraction (regular liquid extraction technique) of the same ambient filter sample (NE sample, blue line) with the molecular fingerprints of the quantifiable OSs of the SPE sample. The circle size represents the OS concentration, determined from the three-dimensional calibration (Fig. S4).

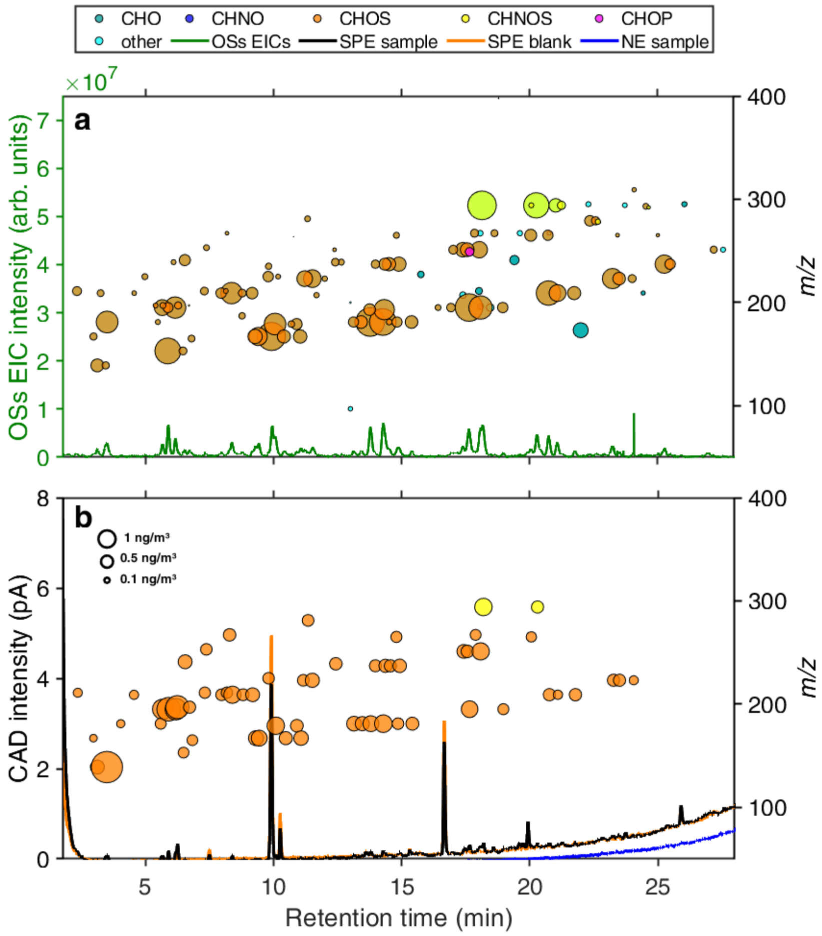

Fig. S13: Comparison of the chromatograms and fingerprints of the enriched OS fraction, measured by UHPLC-HRMS (a) and UHPLC-CAD (b), of a TO filter (TO 2, 24.1.2022 night-time). The x-axis, displaying the retention time (min), is zoomed in to 2.5-28 min of a total method duration of 40 min. There was no integration possible for OS CAD peaks outside of this time window. **(a)** UHPLC-HRMS data of the enriched OS fraction: RT-*m/z* plot (fingerprints, right y-axis) and the sum of the extracted ion chromatograms (EIC, green line, left y-axis) of all the OS fingerprints appearing in the RT-*m/z* plot. The circle size represents the OS intensity. **(b)** Comparison of the UHPLC-CAD chromatograms of the enriched OS fraction (second SPE elution) of the ambient filter sample (SPE sample, black line), the enriched OS fraction of the according ambient filter blank (SPE blank, orange line), and the native extraction (regular liquid extraction technique) of the same ambient filter sample (NE sample, blue line) with the molecular fingerprints of the quantifiable OSs of the SPE sample. The circle size represents the OS concentration, determined from the three-dimensional calibration (Fig. S4).

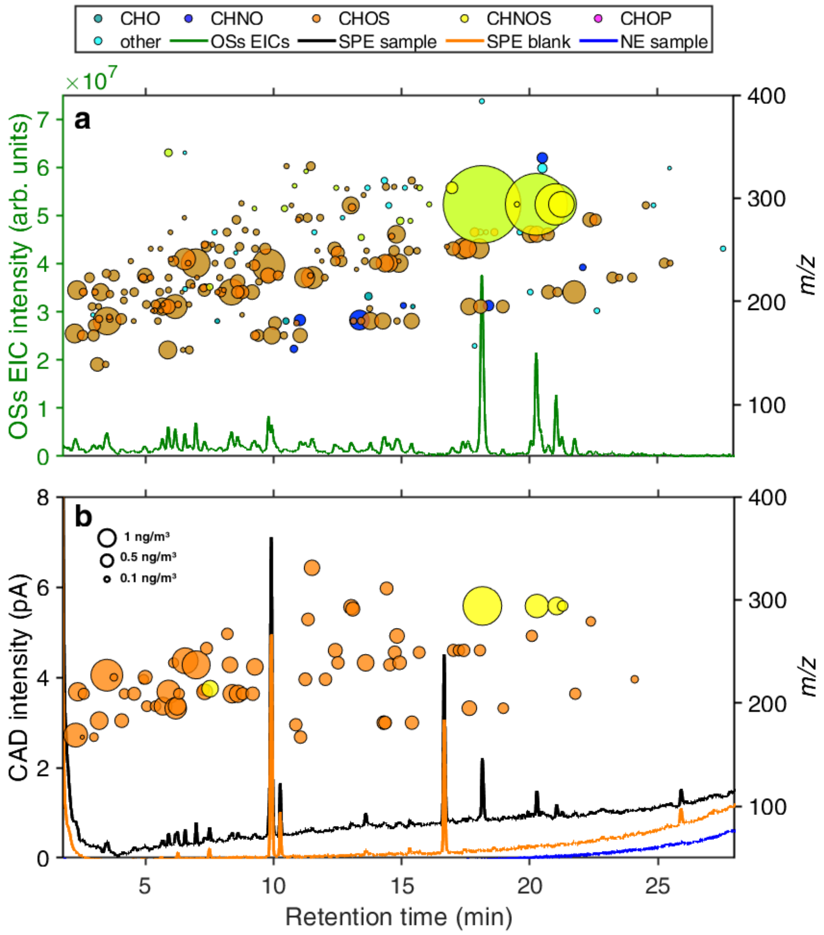

Fig. S14: Comparison of the chromatograms and fingerprints of the enriched OS fraction, measured by UHPLC-HRMS (a) and UHPLC-CAD (b), of a TO filter (TO 3, 28.02.2022 night-time). The x-axis, displaying the retention time (min), is zoomed in to 2.5-28 min of a total method duration of 40 min. There was no integration possible for OS CAD peaks outside of this time window. **(a)** UHPLC-HRMS data of the enriched OS fraction: RT-*m/z* plot (fingerprints, right y-axis) and the sum of the extracted ion chromatograms (EIC, green line, left y-axis) of all the OS fingerprints appearing in the RT-*m/z* plot. The circle size represents the OS intensity. **(b)** Comparison of the UHPLC-CAD chromatograms of the enriched OS fraction (second SPE elution) of the ambient filter sample (SPE sample, black line), the enriched OS fraction of the according ambient filter blank (SPE blank, orange line), and the native extraction (regular liquid extraction technique) of the same ambient filter sample (NE sample, blue line) with the molecular fingerprints of the quantifiable OSs of the SPE sample. The circle size represents the OS concentration, determined from the three-dimensional calibration (Fig. S4).

Figure S15. Correlation of UHPLC-CAD and HRMS chromatograms

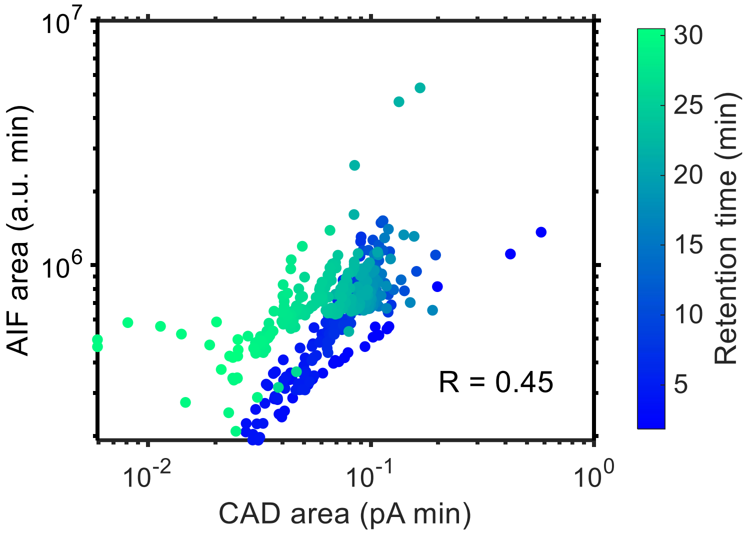

Fig. S15: Correlation of blank-subtracted UHPLC-CAD and UHPLC-HRMS chromatograms (OS ions HSO_4^-, HSO_3^-, $SO_4{\bullet}^-$, and $SO_3{\bullet}^-$ in AIF mode) of an exemplary Handan filter (18.10.2018 daytime). We averaged the data points of each chromatogram in 0.05-minute intervals and use the signal intensity of AIF (a.u.) and CAD (pA) multiplied by the time step (0.05, min), respectively, to get the area of each retention time for both chromatograms. The color bar represents the chromatographic retention time (min).

Figure S16. Backward trajectories

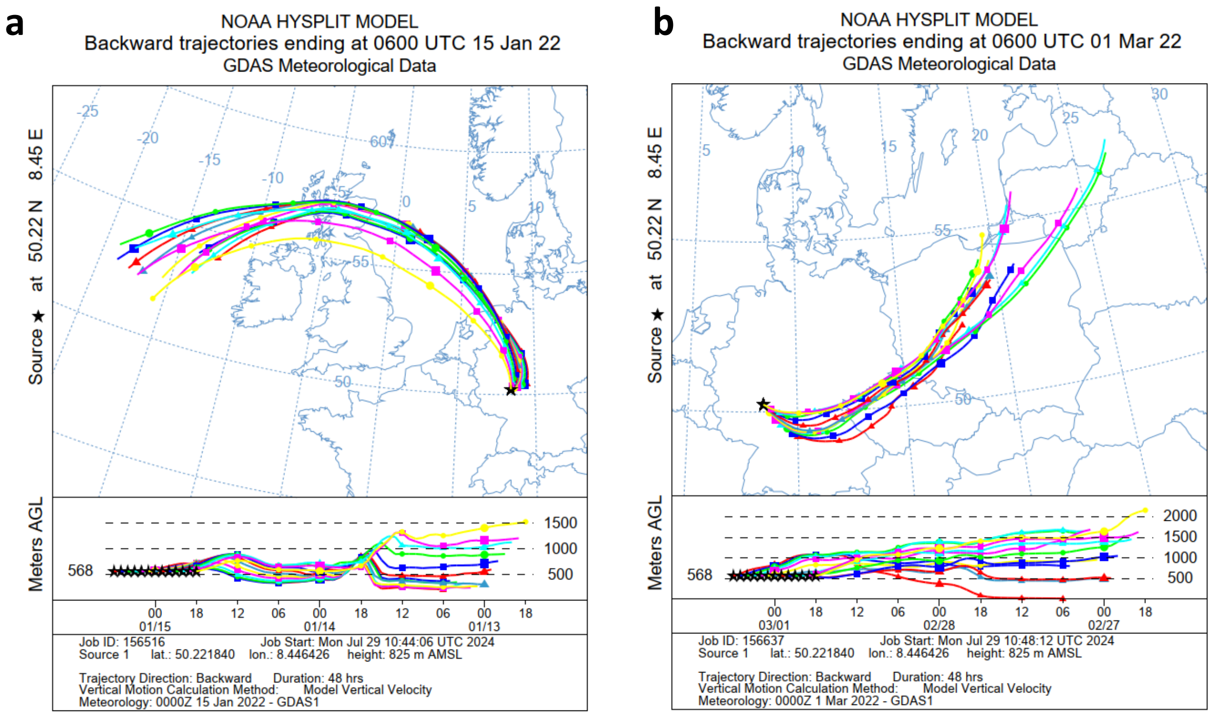

Fig. S16: Backward trajectories for samples TO-2 (a) and TO-3 (b). Backward trajectories for Taunus Observatory (825 m.a.s.l.) starting every hour for the 12-hour filter sampling duration. Duration of backward trajectories is 48 hours and was calculated based on the online web version of the NOAA Hysplit Model [1].

Figure S17. SPE-recovery of isoprene-derived OSs

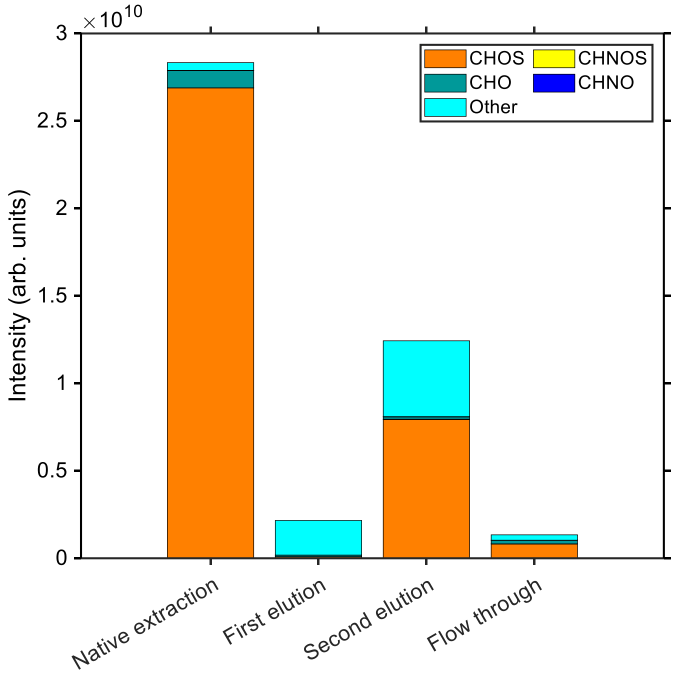

Fig. S17: Summed peak areas of different compound classes in the isoprene-derived OS sample across the native extraction, first elution, second elution, and flow-through, analyzed using the HILIC column.

We conducted a PAM chamber experiment to generate isoprene derived OSs. Using a PAM-OFR, isoprene was oxidized by O_3 (2 ppm) and photooxidation (OH was generated by UV light (λ = 254 nm) inside the OFR). Additionally, we inject SO_2 (55 ppb) at relative humidity ~60%. The SOA exiting the OFR passed through two 50 cm denuders filled with charcoal to remove reactive gas-phase compounds. SOA particles were collected on glass fiber filters at a flow rate of 3 L min^{-1} over 120 minutes. We extracted the filters and applied the SPE method described in the paper, using a HILIC column for the separation of isoprene OSs.

Figure S18. Schematic of the filter preparation

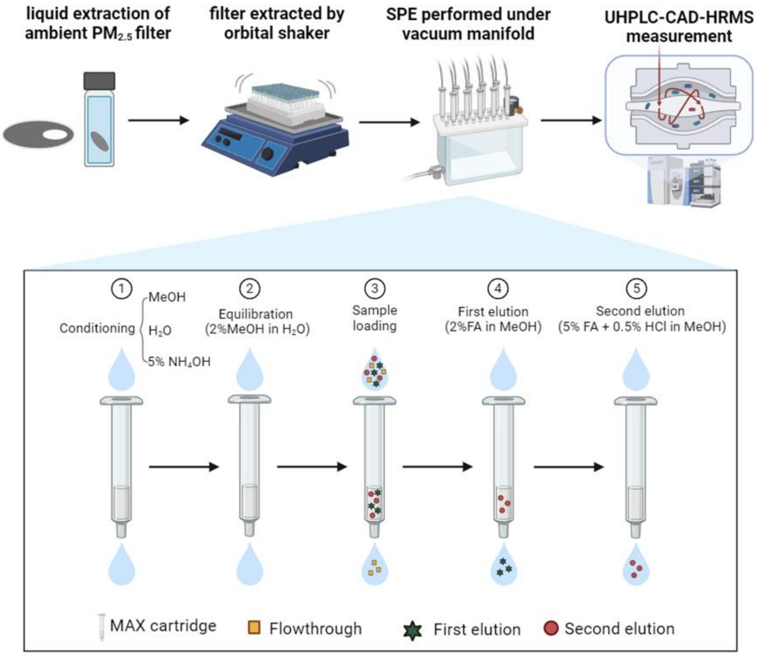

Fig. S18: Workflow of the filter preparation, SPE separation, and UHPLC-CAD-HRMS measurement. Created in BioRender. Ma, J. (2025) https://BioRender.com/r78h920.

Tables S1-6 Quantitive numbers of all OSs in measured samples

Table S1: Quantification of all OSs in the Handan 1 ambient filter sample (18.10.2018, daytime) sorted by ascending retention time. The chemical formula, retention time (RT), mass-to-charge ratio (*m/z*), and OS concentration (ng/m^3) are given for each OS.

RT (min)	Chemical formula	*m/z*	Conc. ng/m^3
1.92	C3 H6 O5 S	152.9861	0.01
1.97	C5 H8 O6 S	194.9968	0.59
2.01	C4 H8 O7 S	198.9917	0.01
2.09	C H N O3 S	105.9603	1.07
2.24	C3 H6 O6 S	168.9811	3.55
2.32	C5 H8 O7 S	210.9916	3.05
2.37	C5 H8 O7 S	210.9916	0.20
2.54	C4 H8 O5 S	167.0019	0.30
2.61	C4 H8 O5 S	167.0019	1.18
2.70	C4 H10 O5 S	169.0176	0.45
2.96	C4 H8 O5 S	167.0019	0.21
3.00	C6 H10 O7 S	225.0074	0.19
3.08	C3 H8 O4 S	139.0070	1.08
3.17	C4 H8 O6 S	182.9968	3.19
3.48	C5 H10 O5 S	181.0177	1.69
4.06	C4 H8 O6 S	182.9967	1.43
4.14	C7 H6 O5 S	200.9863	3.15
4.43	C6 H10 O6 S	209.0124	2.18
4.55	C6 H10 O6 S	209.0124	1.98
4.91	C7 H12 O6 S	223.0281	0.57
4.97	C6 H12 O8 S	225.0073	0.97
5.03	C5 H10 O6 S	197.0125	2.87
5.39	C5 H10 O6 S	197.0125	3.30
5.59	C5 H12 O5 S	183.0330	0.57
5.67	C6 H12 O5 S	195.0331	0.77
5.90	C6 H12 O6 S	211.0281	1.32
5.97	C6 H10 O8 S	241.0025	0.40
6.09	C7 H12 O7 S	239.0231	3.42
6.16	C6 H12 O5 S	195.0333	1.83
6.37	C8 H14 O9 S	285.0283	0.39
6.67	C5 H10 O6 S	197.0125	0.45
6.74	C8 H10 O5 S	217.0174	2.46
6.83	C8 H10 O5 S	217.0174	1.38
7.30	C7 H12 O8 S	255.0180	5.43
7.37	C8 H14 O7 S	253.0389	2.32
7.50	C10 H16 O7 S	279.0543	2.09
7.59	C7 H12 O8 S	255.0182	1.23
7.97	C8 H14 O7 S	253.0389	1.32
8.08	C6 H12 O6 S	211.0280	0.56
8.19	C9 H16 O7 S	267.0543	0.56
8.37	C7 H14 O5 S	209.0488	1.08

8.43	C9 H7 N O5 S	239.9972	0.56
8.58	C7 H14 O6 S	225.0437	0.68
8.67	C8 H14 O7 S	253.0387	1.07
8.83	C8 H14 O7 S	253.0387	0.97
8.89	C8 H16 O6 S	239.0596	2.04
9.08	C9 H16 O7 S	267.0544	0.87
9.15	C7 H14 O5 S	209.0488	1.50
9.25	C9 H18 O7 S	269.0700	0.96
9.30	C9 H16 O7 S	267.0544	0.99
9.47	C8 H14 O7 S	253.0387	0.75
9.66	C9 H16 O6 S	251.0595	0.83
9.79	C7 H14 O6 S	225.0438	4.28
10.17	C7 H14 O6 S	225.0438	1.39
10.56	C11 H18 O9 S	325.0600	0.83
10.63	C9 H16 O8 S	283.0493	0.91
10.83	C9 H10 O6 S	245.0123	0.86
11.02	C5 H12 O4 S	167.0383	3.12
11.11	C10 H18 O8 S	297.0648	0.59
11.33	C6 H11 N O7 S	240.0184	3.28
11.44	C7 H14 O6 S	225.0438	0.67
11.49	C8 H16 O5 S	223.0645	0.66
11.57	C9 H16 O7 S	267.0543	0.68
11.64	C10 H18 O7 S	281.0700	0.88
11.80	C10 H18 O7 S	281.0700	0.70
11.89	C8 H16 O6 S	239.0596	1.57
12.01	C9 H18 O6 S	253.0751	0.51
12.06	C8 H17 N O8 S	286.0602	0.67
12.27	C8 H16 O5 S	223.0645	0.67
12.32	C7 H13 N O9 S	286.0239	0.54
12.39	C8 H16 O6 S	239.0596	1.16
12.53	C10 H18 O8 S	297.0648	0.61
12.59	C8 H18 O5 S	225.0802	0.60
12.64	C8 H16 O6 S	239.0595	1.04
12.81	C8 H16 O6 S	239.0596	0.60
13.01	C11 H18 O7 S	293.0702	0.81
13.17	C10 H18 O8 S	297.0650	0.63
13.39	C8 H9 N O7 S	262.0026	4.07
13.46	C6 H14 O3 S	165.0590	0.69
13.62	C8 H16 O6 S	239.0596	0.48
13.83	C6 H14 O4 S	181.0541	0.64
14.24	C6 H14 O4 S	181.0540	0.84
14.39	C11 H20 O8 S	311.0806	0.77
14.45	C11 H20 O7 S	295.0855	0.57
14.49	C6 H14 O4 S	181.0541	0.63
14.71	C11 H20 O8 S	311.0806	0.85
14.79	C10 H18 O6 S	265.0751	0.53
14.84	C9 H18 O5 S	237.0803	0.60

14.88	C9 H16 O6 S	251.0595	0.53
15.27	C10 H20 O6 S	249.0801	0.42
15.36	C6 H14 O4 S	181.0541	1.13
15.42	C9 H16 O6 S	251.0594	0.67
15.65	C9 H18 O6 S	253.0751	3.66
16.19	C10 H20 O7 S	283.0857	0.71
16.39	C9 H18 O6 S	253.0752	0.60
16.43	C9 H18 O6 S	253.0751	0.51
17.38	C10 H20 O5 S	251.0959	1.01
18.13	C10 H17 N O7 S	294.0656	5.54
18.29	C10 H11 N O7 S	288.0185	0.37
18.63	C10 H20 O6 S	267.0909	0.98
18.76	C8 H16 O3 S	191.0748	0.89
18.94	C7 H16 O4 S	195.0694	0.82
19.29	C8 H16 O4 S	207.0696	1.34
19.83	C10 H18 O6 S	265.0754	0.62
20.06	C11 H22 O5 S	265.1116	1.40
20.27	C10 H17 N O7 S	294.0656	1.48
20.43	C10 H17 N O7 S	294.0656	0.59
20.53	C14 H24 O5 S	303.1276	1.17
20.60	C11 H22 O6 S	281.1066	0.74
20.72	C11 H22 O5 S	265.1117	0.46
21.02	C10 H17 N O7 S	294.0656	0.69
21.24	C10 H17 N O7 S	294.0656	0.31
21.72	C8 H18 O4 S	209.0854	5.46
22.37	C12 H24 O5 S	279.1273	0.88
22.60	C12 H24 O5 S	279.1273	0.63
22.96	C12 H24 O5 S	279.1273	0.51
23.12	C12 H24 O5 S	279.1273	0.46
23.24	C13 H24 O7 S	323.1171	0.39
24.04	C9 H20 O4 S	223.1011	0.58
24.30	C13 H26 O5 S	293.1431	1.66
24.58	C13 H26 O5 S	293.1431	0.40
24.87	C13 H26 O5 S	293.1431	0.39
25.87	C13 H26 O5 S	293.1431	0.43
25.94	C10 H22 O4 S	237.1167	0.10
26.55	C14 H28 O5 S	307.1587	0.26
27.58	C11 H24 O4 S	251.1323	0.37

Table S2: Quantification of all OSs in the Handan 2 ambient filter sample (21.10.2018, daytime) sorted by ascending retention time. The chemical formula, retention time (RT), mass-to-charge ratio (*m/z*), and OS concentration (ng/m^3) are given for each OS.

RT (min)	Chemical formula	*m/z*	Conc. ng/m^3
1.97	C3 H6 O5 S	168.8910	0.73
2.00	C4 H8 O7 S	198.9917	0.42
2.09	C H N O3 S	105.9603	0.14
2.25	C3 H6 O6 S	168.8910	6.42
2.34	C5 H8 O7 S	210.9916	1.74
2.59	C4 H8 O5 S	167.0019	0.55
2.67	C4 H10 O5 S	169.0176	1.01
2.99	C6 H10 O7 S	225.0074	0.06
3.11	C3 H8 O4 S	139.0070	1.04
3.19	C4 H8 O6 S	182.9968	2.43
3.48	C5 H10 O5 S	181.0177	1.50
3.60	C4 H10 O6 S	185.0125	0.34
4.06	C4 H8 O6 S	182.9967	1.65
4.15	C7 H6 O5 S	200.9863	6.29
4.37	C6 H12 O7 S	227.0231	0.47
4.45	C6 H10 O6 S	209.0124	1.28
4.54	C3 H7 N O7 S	199.9869	3.96
4.61	C6 H10 O6 S	209.0124	0.73
4.77	C7 H12 O9 S	271.0127	0.27
4.89	C7 H12 O6 S	223.0281	0.94
5.01	C6 H12 O8 S	225.0073	2.18
5.05	C5 H10 O6 S	197.0125	1.71
5.27	C5 H8 O6 S	194.9968	0.44
5.39	C5 H10 O6 S	197.0124	2.26
5.46	C5 H10 O5 S	181.0177	0.51
5.57	C5 H12 O5 S	183.0332	0.29
5.65	C6 H12 O5 S	195.0331	0.44
5.69	C6 H14 O5 S	197.0489	0.84
5.76	C7 H12 O8 S	255.0182	0.42
5.90	C6 H12 O6 S	211.0281	1.59
5.96	C6 H10 O8 S	241.0025	1.40
6.09	C7 H12 O7 S	239.0231	2.53
6.17	C6 H12 O5 S	195.0333	3.39
6.38	C7 H14 O7 S	241.0386	0.36
6.51	C9 H14 O8 S	281.0335	0.46
6.62	C7 H12 O7 S	239.0230	0.46
6.74	C8 H10 O5 S	217.0174	2.94
6.84	C8 H8 O5 S	215.0019	2.31
6.98	C8 H14 O6 S	237.0437	0.44
7.02	C7 H12 O7 S	239.0231	0.45
7.30	C7 H12 O8 S	255.0181	3.81
7.37	C8 H14 O7 S	253.0389	1.89
7.50	C8 H16 O7 S	255.0544	2.52

7.59	C7 H12 O8 S	255.0181	1.90
7.94	C8 H14 O7 S	253.0389	0.50
8.05	C8 H8 O5 S	215.0018	1.03
8.19	C9 H16 O7 S	267.0543	0.79
8.36	C7 H14 O5 S	209.0488	1.13
8.58	C7 H14 O6 S	225.0437	1.33
8.68	C8 H14 O7 S	253.0387	1.73
8.79	C7 H8 O4 S	187.0070	0.99
8.89	C6 H14 O5 S	197.0489	2.63
9.08	C9 H16 O7 S	267.0544	0.97
9.16	C9 H18 O7 S	269.0700	1.51
9.30	C9 H16 O7 S	267.0544	2.07
9.65	C9 H16 O6 S	251.0595	0.66
9.78	C7 H14 O6 S	225.0438	5.56
10.06	C7 H12 O6 S	179.0384	1.11
10.17	C7 H14 O6 S	225.0438	2.17
10.38	C8 H16 O5 S	223.0645	1.84
10.85	C8 H16 O5 S	223.0645	0.63
11.03	C5 H12 O4 S	167.0383	1.93
11.10	C10 H18 O7 S	281.0700	0.66
11.31	C6 H11 N O7 S	240.0184	2.86
11.58	C9 H16 O7 S	267.0544	0.51
11.65	C10 H18 O7 S	281.0700	0.63
11.80	C10 H18 O7 S	281.0700	1.07
11.86	C8 H16 O6 S	239.0596	0.68
12.40	C8 H16 O6 S	239.0596	0.93
12.53	C10 H18 O8 S	297.0648	0.70
12.63	C10 H18 O7 S	281.0700	2.32
12.77	C10 H18 O7 S	281.0700	1.75
13.39	C8 H9 N O7 S	262.0026	2.10
13.67	C12 H10 O5 S	265.0177	0.73
13.71	C9 H9 N O7 S	274.0029	0.79
14.25	C9 H18 O5 S	237.0802	0.78
14.37	C11 H20 O8 S	311.0806	1.13
14.50	C9 H18 O5 S	237.0802	0.65
14.69	C10 H18 O7 S	281.0700	0.74
14.86	C9 H18 O5 S	237.0802	0.85
15.34	C6 H14 O4 S	181.0541	0.70
15.42	C9 H16 O6 S	251.0594	0.81
15.62	C9 H9 N O7 S	274.0028	4.13
16.76	C9 H18 O6 S	253.0751	0.64
17.04	C10 H20 O5 S	251.0959	1.08
17.38	C10 H20 O5 S	251.0957	1.87
17.57	C10 H20 O5 S	251.0958	1.51
17.84	C10 H20 O6 S	267.0908	0.82
18.02	C10 H20 O5 S	251.0959	0.74
18.13	C10 H17 N O7 S	294.0652	3.31

18.23	C10 H20 O6 S	267.0908	0.45
18.59	C10 H20 O6 S	267.0906	1.32
18.95	C7 H16 O4 S	195.0694	1.05
19.61	C10 H20 O6 S	267.0909	0.89
20.03	C11 H22 O5 S	265.1116	1.32
20.27	C10 H17 N O7 S	294.0652	1.37
20.50	C8 H17 N O7 S	270.0656	0.54
20.56	C8 H18 O3 S	193.0905	0.61
20.74	C11 H22 O5 S	265.1117	0.70
21.02	C10 H17 N O7 S	294.0656	0.44
21.12	C11 H22 O6 S	281.1066	0.54
21.73	C8 H18 O4 S	209.0853	2.50
21.93	C16 H22 O8 S	373.0965	0.54
22.35	C12 H24 O5 S	279.1273	1.46
22.58	C12 H24 O5 S	279.1273	0.95
22.95	C12 H24 O5 S	279.1273	0.61
23.11	C12 H24 O5 S	279.1273	0.50
23.21	C9 H20 O4 S	223.1011	0.47
23.48	C9 H20 O4 S	223.1011	1.02
23.68	C9 H20 O4 S	223.1011	0.36
24.00	C9 H20 O4 S	223.1011	0.70
24.55	C13 H26 O5 S	293.1431	0.94
24.85	C13 H26 O5 S	293.1431	0.47
25.06	C13 H26 O6 S	309.1380	0.52
25.26	C10 H22 O4 S	237.1167	0.48
25.87	C13 H26 O6 S	309.1380	0.65
26.27	C14 H28 O5 S	307.1587	0.60
26.53	C14 H28 O5 S	307.1587	0.22
26.77	C14 H28 O5 S	307.1587	0.14
26.95	C11 H24 O4 S	251.1323	0.70
27.56	C11 H24 O4 S	251.1323	0.82
27.70			0.13
27.75			0.12
28.43	C12 H26 O3 S	249.1531	0.75
28.66	C15 H32 O5 S	323.1900	0.05
28.99		168.8910	0.72

Table S3: Quantification of all OSs in the Handan 3 ambient filter sample (21.10.2018, night-time) sorted by ascending retention time. The chemical formula, retention time (RT), mass-to-charge ratio (*m/z*), and OS concentration (ng/m^3) are given for each OS.

RT (min)	Chemical formula	*m/z*	Conc. ng/m^3
2.00	C4 H8 O7 S	198.9917	1.74
2.09	C H N O3 S	105.9603	0.07
2.26	C3 H6 O6 S	168.9811	3.91
2.38	C5 H8 O7 S	210.9916	0.45
2.60	C4 H8 O5 S	167.0019	0.68
2.68	C4 H10 O5 S	169.0176	0.12
2.84	C3 H5 N O6 S	181.9765	0.05
3.09	C6 H10 O7 S	225.0074	0.06
3.19	C4 H8 O6 S	182.9968	1.53
3.48	C5 H10 O7 S	213.0074	0.54
4.08	C4 H8 O6 S	182.9967	9.87
4.16	C7 H6 O5 S	200.9863	0.85
4.49	C6 H6 O3 S	156.9963	3.26
4.55	C3 H7 N O7 S	199.9869	1.21
4.91	C6 H10 O7 S	225.0073	0.57
5.03	C6 H6 O4 S	172.9913	1.37
5.05	C6 H10 O6 S	209.0125	1.02
5.28	C5 H8 O6 S	194.9968	0.77
5.39	C5 H10 O6 S	197.0124	1.36
5.62	C6 H12 O5 S	195.0331	0.32
5.68	C7 H12 O7 S	239.0232	0.55
5.80	C8 H9 N O6 S	246.0077	0.30
5.92	C8 H8 O5 S	215.0021	1.80
6.03	C4 H7 N O7 S	211.9871	1.11
6.10	C7 H12 O7 S	239.0231	2.40
6.19	C6 H12 O6 S	211.0281	2.31
6.26	C6 H14 O5 S	197.0489	1.19
6.49	C7 H6 O4 S	184.9914	0.38
6.55	C7 H14 O7 S	241.0388	0.40
6.69	C8 H8 O4 S	199.0070	0.48
6.74	C8 H10 O5 S	217.0174	0.76
6.85	C8 H8 O5 S	215.0019	2.56
7.02	C6 H13 N O8 S	258.0287	0.41
7.15	C6 H11 N O8 S	256.0133	0.79
7.30	C6 H12 O6 S	211.0282	2.37
7.38	C8 H14 O7 S	253.0389	1.64
7.51	C10 H16 O7 S	279.0543	3.60
7.60	C7 H12 O8 S	255.0179	2.36
7.88	C9 H16 O6 S	251.0594	0.45
7.95	C6 H13 N O8 S	258.0288	0.54
8.06	C6 H12 O6 S	211.0280	0.74
8.38	C7 H14 O5 S	209.0488	1.14
8.59	C8 H16 O7 S	255.0543	0.74

8.68	C8 H16 O5 S	223.0647	0.63
8.80	C5 H10 Cl N O8 S	277.9742	0.55
8.84	C5 H9 N O7 S	226.0027	0.92
8.88	C8 H14 O8 S	269.0336	0.99
8.93	C8 H16 O6 S	239.0596	0.92
9.10	C10 H16 O9 S	267.0544	0.67
9.17	C7 H14 O5 S	209.0488	1.48
9.29	C9 H10 O5 S	229.0175	1.31
9.58	C7 H16 O5 S	211.0645	0.58
9.67	C9 H16 O6 S	251.0595	0.43
9.81	C7 H13 N O8 S	270.0287	1.86
10.18	C7 H14 O6 S	225.0438	0.86
10.85	C5 H11 N O7 S	228.0184	1.14
11.04	C10 H18 O7 S	281.0700	1.40
11.33	C6 H11 N O7 S	240.0184	2.70
12.56	C10 H18 O8 S	297.0649	0.57
12.59	C8 H16 O6 S	239.0595	0.55
12.66	C8 H16 O5 S	223.0645	0.93
12.74	C8 H17 N O8 S	286.0602	0.84
12.82	C8 H16 O6 S	239.0596	0.63
13.03	C9 H18 O7 S	225.0802	0.58
13.15	C10 H18 O8 S	297.0650	0.93
13.21	C7 H13 N O9 S	286.0240	0.55
13.39	C8 H9 N O7 S	262.0026	5.71
13.47	C8 H16 O6 S	239.0596	0.81
13.68	C12 H10 O5 S	265.0177	1.55
13.99	C9 H18 O5 S	237.0802	0.46
14.40	C11 H20 O8 S	311.0806	1.00
15.41	C9 H16 O6 S	251.0594	0.89
15.59	C9 H9 N O7 S	274.0028	7.43
16.18	C9 H18 O5 S	237.0802	0.62
16.66	C13 H12 O5 S	279.0333	3.21
16.77	C9 H18 O6 S	253.0751	0.89
17.01	C10 H11 N O7 S	288.0183	0.59
17.42	C7 H16 O3 S	179.0747	2.14
17.58	C7 H16 O4 S	195.0696	1.52
17.75	C11 H20 O5 S	263.0957	1.38
17.88	C10 H20 O6 S	267.0908	1.02
17.95	C7 H12 O9 S	271.0127	0.96
18.03	C10 H20 O5 S	251.0959	0.56
18.12	C10 H17 N O7 S	294.0652	6.62
18.45	C10 H20 O6 S	267.0908	0.40
18.55	C10 H20 O6 S	267.0908	0.39
18.78	C8 H16 O3 S	191.0748	0.72
18.95	C7 H16 O4 S	195.0697	0.55
19.27	C7 H15 N O7 S	256.0497	0.70
20.04	C11 H22 O5 S	265.1116	0.83

20.10	C10 H17 N O7 S	294.0656	0.42
20.27	C10 H17 N O7 S	294.0656	1.91
20.46	C10 H17 N O7 S	294.0656	0.74
20.52	C11 H22 O6 S	281.1066	0.75
20.56	C12 H22 O5 S	277.1117	1.49
20.70	C8 H18 O4 S	209.0854	0.58
21.03	C10 H17 N O7 S	294.0656	0.89
21.11	C11 H22 O6 S	281.1066	0.60
21.27	C10 H17 N O7 S	294.0656	0.46
21.73	C8 H18 O4 S	209.0853	3.25
21.84	C10 H19 N O9 S	328.0711	0.37
22.09	C8 H17 N O7 S	270.0654	0.36
22.37	C10 H22 O5 S	253.1116	0.77
22.59	C12 H24 O5 S	279.1273	0.71
22.97	C12 H24 O5 S	279.1273	0.33
23.59	C9 H20 O4 S	223.1011	0.60
23.67	C9 H20 O4 S	223.1010	0.26
24.04	C9 H20 O4 S	223.1010	0.37
24.30	C10 H22 O4 S	237.1167	1.52
24.59	C13 H26 O5 S	293.1431	0.47
24.86	C13 H26 O5 S	293.1431	0.26
25.28	C10 H22 O4 S	237.1167	0.39
25.88	C10 H22 O4 S	237.1167	0.40
25.96	C14 H28 O6 S	323.1536	0.26
26.01	C10 H21 N O7 S	298.0967	0.24
26.06			0.26
26.11	C12 H26 O5 S	281.1430	0.14
27.30			1.45
27.58	C11 H24 O4 S	251.1324	0.51
28.01	C15 H30 O5 S	321.1743	0.34
28.22	C14 H30 O4 S	293.1794	0.19

Table S4: Quantification of all OSs in the Taunus Observatory (TO) 1 ambient filter sample (11.12.2021, daytime) sorted by ascending retention time. The chemical formula, retention time (RT), mass-to-charge ratio (*m/z*), and OS concentration (ng/m^3) are given for each OS.

RT (min)	Chemical formula	*m/z*	Conc. ng/m^3
2.28	C3 H6 O6 S	168.9813	1.13
2.36	C5 H8 O7 S	210.9918	1.10
2.59	C4 H8 O5 S	167.0019	0.40
3.02	C4 H8 O5 S	167.0020	0.29
3.24	C6 H10 O6 S	209.0124	1.10
3.49	C5 H10 O5 S	181.0175	0.69
3.67	C7 H10 O8 S	253.0023	0.08
3.77	C6 H10 O7 S	225.0074	0.27
4.10	C4 H8 O6 S	182.9968	0.37
4.18	C6 H10 O6 S	209.0125	0.43
4.56	C6 H10 O6 S	209.0123	0.54
4.93	C7 H12 O6 S	223.0281	0.89
4.99	C6 H10 O7 S	225.0073	0.58
5.06	C5 H10 O6 S	197.0124	0.37
5.41	C5 H10 O6 S	197.0123	0.32
5.43	C7 H12 O6 S	223.0280	0.36
5.46	C7 H12 O6 S	223.0280	0.27
5.56	C6 H10 O6 S	209.0124	0.28
5.67	C6 H12 O5 S	195.0331	0.35
5.90	C10 H19 N O10 S	344.0657	0.91
5.96	C7 H14 O7 S	223.0280	0.36
6.12	C7 H12 O7 S	239.0228	0.49
6.19	C6 H12 O5 S	195.0332	0.66
6.56	C7 H14 O7 S	241.0387	1.46
6.69	C5 H10 O6 S	197.0122	0.36
6.85	C6 H13 N O8 S	258.0288	0.38
6.98	C9 H14 O8 S	281.0334	1.02
7.17	C7 H8 O3 S	171.0122	0.43
7.31	C9 H14 O8 S	281.0335	0.91
7.39	C8 H14 O7 S	253.0386	0.82
7.52	C7 H12 O6 S	223.0282	0.42
7.57	C10 H16 O7 S	279.0542	0.65
7.62	C7 H12 O8 S	255.0180	0.51
7.85	C9 H16 O6 S	251.0594	0.40
7.91	C8 H12 O8 S	223.0282	0.61
7.96	C7 H14 O8 S	257.0335	0.58
8.30	C10 H16 O7 S	279.0543	0.77
8.40	C7 H14 O5 S	209.0488	0.66
8.61	C7 H14 O5 S	209.0488	0.60
8.68	C8 H14 O7 S	253.0386	0.45
8.91	C8 H14 O8 S	269.0336	0.58
9.19	C7 H14 O5 S	209.0487	0.57
9.24	C9 H14 O8 S	281.0336	0.44

9.47	C9 H14 O5 S	233.0488	0.52
9.52	C9 H18 O7 S	269.0702	0.52
9.69	C9 H16 O6 S	251.0593	0.53
10.11	C7 H14 O6 S	225.0438	0.63
10.84	C9 H15 N O9 S	312.0393	0.61
11.06	C5 H12 O4 S	167.0382	0.74
12.40	C9 H16 O6 S	251.0594	0.73
12.56	C10 H14 O6 S	261.0437	0.50
13.04	C11 H18 O7 S	293.0701	0.54
13.09	C7 H15 N O4 S	208.0648	0.58
13.15	C7 H13 N O9 S	242.0340	0.78
14.82	C10 H18 O6 S	265.0752	1.13
15.41	C6 H14 O4 S	181.0539	0.86
17.00	C10 H17 N O8 S	310.0601	0.68
18.15	C10 H17 N O7 S	294.0653	6.07
20.28	C10 H17 N O7 S	294.0654	2.35
21.05	C10 H17 N O7 S	294.0654	0.72
21.28	C10 H17 N O7 S	294.0654	0.47
21.77	C8 H18 O4 S	209.0852	1.10

Table S5: Quantification of all OSs in the Taunus Observatory (TO) 2 ambient filter sample (14.01.2022, night-time) sorted by ascending retention time. The chemical formula, retention time (RT), mass-to-charge ratio (*m/z*), and OS concentration (ng/m³) are given for each OS.

RT (min)	Chemical formula	*m/z*	Conc. ng/m^3
2.28			0.11
2.36	C5 H8 O7 S	210.9918	0.29
2.59			0.12
2.97	C4 H8 O5 S	167.0020	0.20
3.12	C3 H8 O4 S	139.0070	0.67
3.50	C3 H8 O4 S	139.0070	3.31
4.04	C5 H10 O5 S	181.0175	0.23
4.55	C6 H10 O6 S	209.0123	0.30
5.67	C6 H12 O5 S	195.0331	1.41
5.59	C5 H10 O5 S	181.0176	0.42
5.90	C6 H12 O5 S	195.0332	1.89
6.19	C6 H12 O5 S	195.0332	1.56
6.24	C6 H14 O5 S	197.0488	1.82
6.49	C4 H10 O4 S	153.0225	0.40
6.55	C7 H14 O7 S	241.0387	0.67
6.72	C5 H10 O6 S	197.0122	0.51
6.83	C5 H10 O4 S	165.0226	0.39
7.32	C6 H12 O6 S	211.0281	0.44
7.38	C8 H14 O7 S	253.0386	0.46
7.97	C7 H14 O5 S	209.0487	0.47
8.18	C7 H16 O5 S	211.0643	0.45
8.28	C9 H16 O7 S	267.0543	0.54
8.39	C7 H14 O5 S	209.0488	1.01
8.81	C7 H14 O5 S	209.0489	0.48
9.18	C7 H14 O5 S	209.0487	0.66
9.31	C5 H12 O4 S	167.0383	0.78
9.44	C5 H12 O4 S	167.0383	0.86
9.81	C7 H14 O6 S	225.0438	0.46
10.09	C6 H12 O4 S	179.0384	1.03
10.48	C5 H12 O4 S	167.0382	0.60
10.91	C6 H12 O4 S	179.0384	0.55
11.07	C5 H12 O4 S	167.0382	0.73
11.16	C8 H16 O5 S	223.0644	0.47
11.24			0.51
11.35	C10 H18 O7 S	281.0700	0.47
11.51	C8 H16 O5 S	223.0645	0.70
12.43	C8 H16 O6 S	239.0595	0.53
13.14	C6 H14 O4 S	181.0540	0.74
13.46	C6 H14 O4 S	181.0540	0.69
13.80	C6 H14 O4 S	181.0540	0.86
13.97	C9 H18 O5 S	237.0801	0.49
14.28	C6 H14 O4 S	181.0540	1.06
14.37	C9 H18 O5 S	237.0799	0.60

14.56	C9 H18 O5 S	237.0801	0.51
14.79	C10 H18 O6 S	265.0752	0.43
14.86	C6 H14 O4 S	181.0540	0.46
14.92	C9 H18 O5 S	237.0801	0.62
15.42	C6 H14 O4 S	181.0539	0.58
17.42	C10 H20 O5 S	251.0959	0.65
17.65	C7 H16 O4 S	195.0696	0.95
17.57	C10 H20 O5 S	251.0958	0.55
17.89	C10 H20 O6 S	267.0907	0.40
18.08	C10 H20 O5 S	251.0959	1.01
18.19	C10 H17 N O7 S	294.0653	1.04
18.97	C7 H16 O4 S	195.0696	0.42
19.94	C45 H72 O5 S	723.5033	1.78
20.06	C11 H22 O5 S	265.1115	0.36
20.30	C10 H17 N O7 S	294.0654	0.51
20.77	C8 H18 O4 S	209.0852	0.52
21.10	C8 H18 O4 S	209.0852	0.30
21.78	C8 H18 O4 S	209.0852	0.52
23.27	C9 H20 O4 S	223.1009	0.51
23.50	C9 H20 O4 S	223.1010	0.51
24.06	C9 H20 O4 S	223.1010	0.29

Table S6: Quantification of all OSs in the Taunus Observatory (TO) 3 ambient filter sample (28.02.2022, night-time) sorted by ascending retention time. The chemical formula, retention time (RT), mass-to-charge ratio (*m/z*), and OS concentration (ng/m^3) are given for each OS.

RT (min)	Chemical formula	*m/z*	Conc. ng/m^3
2.28	C3 H6 O6 S	168.9813	1.94
2.36	C5 H8 O7 S	210.9918	1.15
2.54	C4 H8 O5 S	167.0019	0.06
2.59	C6 H10 O6 S	209.0124	0.42
2.99	C4 H8 O5 S	167.0020	0.26
3.19	C4 H8 O6 S	182.9967	1.06
3.49	C6 H12 O7 S	227.0230	3.42
3.77	C6 H10 O7 S	225.0074	0.20
4.07	C4 H8 O6 S	182.9968	0.65
4.18	C6 H10 O6 S	209.0125	0.36
4.56	C6 H10 O6 S	209.0123	0.58
4.93	C7 H12 O6 S	223.0281	0.34
4.99	C6 H10 O7 S	225.0073	0.66
5.06	C5 H10 O6 S	197.0124	0.39
5.39	C5 H10 O6 S	197.0123	0.38
5.68	C6 H14 O5 S	197.0488	1.08
5.90	C6 H12 O6 S	211.0282	1.83
6.10	C7 H12 O7 S	239.0228	0.34
6.18	C6 H12 O5 S	195.0332	1.59
6.24	C6 H14 O5 S	197.0488	1.09
6.31	C7 H14 O5 S	209.0488	0.40
6.55	C7 H14 O7 S	241.0387	2.26
6.98	C8 H14 O6 S	237.0437	2.67
7.27	C8 H6 O5 S	212.9864	0.44
7.31	C6 H12 O6 S	211.0281	0.85
7.38	C8 H14 O7 S	253.0386	0.50
7.52	C4 H9 N O7 S	214.0026	0.94
8.20	C9 H16 O7 S	267.0543	0.45
8.30	C8 H14 O6 S	237.0436	0.83
8.39	C7 H14 O5 S	209.0488	1.16
8.61	C7 H14 O5 S	209.0488	1.04
8.79	C7 H16 O6 S	209.0488	0.53
9.18	C7 H14 O5 S	209.0487	0.63
9.27	C9 H16 O5 S	235.0647	0.94
10.87	C6 H12 O4 S	179.0384	0.53
11.05	C5 H12 O4 S	167.0382	0.53
11.24	C8 H16 O5 S	223.0644	0.57
11.35	C10 H18 O7 S	281.0700	0.55
11.50	C14 H20 O7 S	331.0855	0.82
12.03	C8 H16 O5 S	223.0645	0.54
12.41	C9 H16 O6 S	251.0594	0.68
12.51	C8 H16 O6 S	239.0595	0.53
13.03	C11 H18 O7 S	293.0701	0.78

13.09	C12 H20 O6 S	291.0908	0.67
13.59	C8 H16 O6 S	239.0595	0.94
14.30	C6 H14 O4 S	181.0540	0.64
14.35	C6 H14 O4 S	181.0540	0.57
14.41	C11 H20 O8 S	311.0808	0.57
14.53	C9 H18 O5 S	237.0801	0.52
14.73	C10 H18 O5 S	249.0801	0.58
14.82	C10 H18 O6 S	265.0752	0.74
14.92	C8 H16 O6 S	239.0593	0.64
15.40	C6 H14 O4 S	181.0539	0.61
15.68	C10 H18 O5 S	249.0801	0.49
17.02	C10 H20 O5 S	251.0958	0.51
17.23	C10 H20 O5 S	251.0959	0.44
17.42	C10 H20 O5 S	251.0958	0.52
17.64	C7 H16 O4 S	195.0696	0.73
18.06	C10 H20 O5 S	251.0959	0.44
18.15	C10 H17 N O7 S	294.0653	5.06
18.97	C7 H16 O4 S	195.0696	0.39
20.08	C11 H22 O5 S	265.1115	0.43
20.28	C10 H17 N O7 S	294.0654	1.86
21.05	C10 H17 N O7 S	294.0654	1.07
21.29	C10 H17 N O7 S	294.0654	0.38
21.78	C8 H18 O4 S	209.0852	0.44
22.38	C12 H24 O5 S	279.1272	0.32
24.10	C9 H20 O4 S	223.1010	0.20

Table S7. Summary of the OS fraction in OM quantified by various techniques

Table S7. Summary of the OS fraction in OM across different locations, quantified by various techniques.

	location	Particle size	OS fraction in OM (%)	Instruments	Reference
**Direct quantification**					
	Beijing, China	$PM_{2.5}$	0.31	HPLC/MS	Wang et al., 2018 [2]
	Shanghai, China	$PM_{2.5}$	0.62	UPLC/ToF-MS	Wang et al., 2021 [3]
	Beijing (Winter), China	$PM_{2.5}$	0.7	UHPLC/Orbitrap-MS	Ma et al., 2022 [4]
	Xi'an (Winter), China	$PM_{2.5}$	1.4	UHPLC/ToF-MS	Glasius et al., 2022 [5]
	4 sites, Asia	$PM_{2.5}$	1.4	UPLC/ ToF-MS	Stone et al., 2012 [6]
	Beijing, China	PM_1	2	FIGAERO ToF-CIMS	Le Breton et al., 2018 [7]
	19 sites, USA	$PM_{2.5}$	3.22	UPLC/ToF-MS	Chen et al., 2021 [8]
	Bohai and Yellow Sea, China	$PM_{2.5}$	3.47	UHPLC/Orbitrap-MS	Wang et al., 2023 [9]
	Beijing (Summer), China	$PM_{2.5}$	4	UHPLC/Orbitrap-MS	Ma et al., 2022 [4]
	Xi'an (Summer), China	$PM_{2.5}$	7	UHPLC/ToF-MS	Glasius et al., 2022 [5]
**Indirect quantification**					
	Fairbanks, USA	$PM_{2.5}$	1.29	XRF, IC	Shakya et al., 2013 [10]
	Hongkong, China	PM_1	5 (Minimum estimation)	HR-ToF-AMS	Huang et al., 2015 [11]
	334 sites, USA	$PM_{2.5}$	7.5	XRF, IC	Shakya et al., 2015 [12]
	12 sites, USA	$PM_{2.5}$	8.12	XRF, IC	Tolocka et al., 2012 [13]
	Riverside, USA	$PM_{1.5}$	12	AMS, IC	Farmer et al., 2010 [14]
	19 sites, USA	$PM_{2.5}$	14.51	ICP−OES, IC	Chen et al., 2021 [8]
	Beijing, China	$PM_{2.5}$	25.05	XRF, IC	He et al., 2001 [15]
	K-puszta, Hungary	$PM_{2.5}$	29	XRF, IC	Lukács et al., 2009 [16]
	K-puszta, Hungary	$PM_{2.5}$	30	XRF, IC	Surratt et al., 2008 [17]

Tables S8-9. Compound Discoverer workflow

Table S8: Compound Discoverer workflow for the UHPLC HRMS sequence.

Processing node 42: Input Files

Input Data:

- File Name(s) (Hidden):

Processing node 2: Select Spectra

----

1. Spectrum Properties Filter:

- Lower RT Limit: 1

- Upper RT Limit: 18.5

- First Scan: 0

- Last Scan: 0

- Ignore Specified Scans: (not specified)

- Lowest Charge State: 0

- Highest Charge State: 0

- Min. Precursor Mass: 50 Da

- Max. Precursor Mass: 5000 Da

- Total Intensity Threshold: 0

- Minimum Peak Count: 1

2. Scan Event Filters:

- Mass Analyzer: Is FTMS

- MS Order: Any

- Activation Type: Is HCD

- Min. Collision Energy: 0

- Max. Collision Energy: 1000

- Scan Type: Any

- Polarity Mode: Is -

- MS1 Mass Range: (not specified)

- FAIMS CV: (not specified)

3. Peak Filters:

- S/N Threshold (FT-only): 5

4. Replacements for Unrecognized Properties:

- Unrecognized Charge Replacements: 1

- Unrecognized Mass Analyzer Replacements: ITMS

- Unrecognized MS Order Replacements: MS2

- Unrecognized Activation Type Replacements: HCD

- Unrecognized Polarity Replacements: -

- Unrecognized MS Resolution@200 Replacements: 60000

- Unrecognized MSn Resolution@200 Replacements: 30000

5. General Settings:

- Precursor Selection: Use MS1 Precursor

- Use Isotope Pattern in Precursor Reevaluation: True

- Provide Profile Spectra: Automatic

- Store Chromatograms: False

Processing node 23: Align Retention Times (ChromAlign)

----

1. General Settings:

- Reference File: 20220805_H_HCl1_neg01

Processing node 24: Detect Compounds

----

1. General Settings:

- Mass Tolerance [ppm]: 4 ppm

- Min. Peak Intensity: 10000

- Min. # Scans per Peak: 5

- Use Most Intense Isotope Only: False

2. Trace Detection:

- Max. Number of Gaps to Correct: 2

- Min. Number of Adjacent Non-Zeros: 2

3. Peak Detection:

- Chromatographic S/N Threshold: 1.5

- Remove Baseline: False

- Gap Ratio Threshold: 0.35

- Max. Peak Width [min]: 0.2

- Min. Relative Valley Depth: 0.1

4. Isotope Pattern Detection:

- Group Isotopes for: Br; Cl

- Use Peak Quality for Isotope Grouping: True

- Filter out Features with Bad Peaks Only: True

- Zig-Zag Index Threshold: 0.2

- Jaggedness Threshold: 0.4

- Modality Threshold: 0.9

- Remove Potentially False Positive Isotopes: True

5. Compound Detection:

- Ions: [2M-H]-1; [M-H]-1; [M-H-H2O]-1

- Base Ions: [M-H]-1

- Remove Singlets: True

6. AcquireX Settings:

- Detect Persistent Background Ions: False

Processing node 4: Group Compounds

----

1. General Settings:

- Mass Tolerance: 2 ppm

- RT Tolerance [min]: 0.1

- Align Peaks: False

- Preferred Ions: [M-H]-1

- Area Integration: Most Common Ion

2. Peak Rating Contributions:

- Area Contribution: 3

- CV Contribution: 1

- FWHM to Base Contribution: 5

- Jaggedness Contribution: 5

- Modality Contribution: 5

- Zig-Zag Index Contribution: 5

3. Peak Rating Filter:

- Peak Rating Threshold: 3

- Number of Files: 1

Processing node 41: Fill Gaps

----

1. General Settings:

- Mass Tolerance: 2 ppm

- S/N Threshold: 1.5

- Use Real Peak Detection: True

- Apply Restrictive Gap Filling: True

Processing node 18: Mark Background Compounds

----

1. General Settings:

- Max. Sample/Blank: 5

- Max. Blank/Sample: 0

- Hide Background: True

Processing node 11: Assign Compound Annotations

----

1. General Settings:

- Mass Tolerance: 1 ppm

2. Data Sources:

- Data Source #1: Predicted Compositions

- Data Source #2: mzCloud Search

- Data Source #3: ChemSpider Search

- Data Source #4: (not specified)

- Data Source #5: (not specified)

- Data Source #6: (not specified)

- Data Source #7: (not specified)

3. Scoring Rules:

- Use mzLogic: True

- Use Spectral Distance: True

- SFit Threshold: 20

- SFit Range: 20

4. Reprocessing:

- Clear Names: False

Processing node 38: Search mzCloud

----

1. General Settings:

- Compound Classes: All

- Precursor Mass Tolerance: 10 ppm

- FT Fragment Mass Tolerance: 10 ppm

- IT Fragment Mass Tolerance: 0.4 Da

- Library: Autoprocessed; Reference

- Post Processing: Recalibrated

- Max. # Results: 10

- Annotate Matching Fragments: False

- Search MSn Tree: False

2. DDA Search:

- Identity Search: HighChem HighRes

- Match Activation Type: True

- Match Activation Energy: Match with Tolerance

- Activation Energy Tolerance: 20

- Apply Intensity Threshold: True

- Similarity Search: None

- Match Factor Threshold: 60

3. DIA Search:

- Use DIA Scans for Search: False
- Max. Isolation Width [Da]: 500
- Match Activation Type: False
- Match Activation Energy: Any
- Activation Energy Tolerance: 100
- Apply Intensity Threshold: False
- Match Factor Threshold: 20

Processing node 5: Predict Compositions

----

1. Prediction Settings:

- Mass Tolerance: 2 ppm
- Min. Element Counts: C H
- Max. Element Counts: C90 H190 Br3 Cl4 N4 O20 P S3
- Min. RDBE: 0
- Max. RDBE: 40
- Min. H/C: 0.1
- Max. H/C: 3.5
- Max. # Candidates: 10
- Max. # Internal Candidates: 200

2. Pattern Matching:

- Intensity Tolerance [%]: 30
- Intensity Threshold [%]: 0.1
- S/N Threshold: 5
- Min. Spectral Fit [%]: 30
- Min. Pattern Cov. [%]: 90
- Use Dynamic Recalibration: True

3. Fragments Matching:

- Use Fragments Matching: True
- Mass Tolerance: 1 ppm

- S/N Threshold: 5

Processing node 34: Merge Features

----

1. Peak Consolidation:

- Mass Tolerance: 2 ppm

- RT Tolerance [min]: 0.05

Table S9. CD-workflow for the UHPLC-CAD-HRMS sequence

Processing node 42: Input Files

Input Data:

- File Name(s) (Hidden):

Processing node 2: Select Spectra

----

1. Spectrum Properties Filter:

- Lower RT Limit: 1

- Upper RT Limit: 38.5

- First Scan: 0

- Last Scan: 0

- Ignore Specified Scans: (not specified)

- Lowest Charge State: 0

- Highest Charge State: 0

- Min. Precursor Mass: 50 Da

- Max. Precursor Mass: 5000 Da

- Total Intensity Threshold: 0

- Minimum Peak Count: 1

2. Scan Event Filters:

- Mass Analyzer: Is FTMS

- MS Order: Any

- Activation Type: Is HCD

- Min. Collision Energy: 0

- Max. Collision Energy: 1000

- Scan Type: Any

- Polarity Mode: Is -

- MS1 Mass Range: (not specified)

- FAIMS CV: (not specified)

3. Peak Filters:

- S/N Threshold (FT-only): 5

4. Replacements for Unrecognized Properties:

- Unrecognized Charge Replacements: 1

- Unrecognized Mass Analyzer Replacements: ITMS

- Unrecognized MS Order Replacements: MS2

- Unrecognized Activation Type Replacements: HCD

- Unrecognized Polarity Replacements: -

- Unrecognized MS Resolution@200 Replacements: 60000

- Unrecognized MSn Resolution@200 Replacements: 30000

5. General Settings:

- Precursor Selection: Use MS1 Precursor

- Use Isotope Pattern in Precursor Reevaluation: True

- Provide Profile Spectra: Automatic

- Store Chromatograms: False

Processing node 23: Align Retention Times (ChromAlign)

----

1. General Settings:

- Reference File: 20231120_40RT_ambient_SPE_Handan_20181018D_CAD25_neg00

Processing node 24: Detect Compounds

----

1. General Settings:

- Mass Tolerance [ppm]: 4 ppm

- Min. Peak Intensity: 10000

- Min. # Scans per Peak: 5

- Use Most Intense Isotope Only: False

2. Trace Detection:

- Max. Number of Gaps to Correct: 2

- Min. Number of Adjacent Non-Zeros: 2

3. Peak Detection:

- Chromatographic S/N Threshold: 1.5

- Remove Baseline: False

- Gap Ratio Threshold: 0.35

- Max. Peak Width [min]: 0.2

- Min. Relative Valley Depth: 0.1

4. Isotope Pattern Detection:

- Group Isotopes for: Br; Cl

- Use Peak Quality for Isotope Grouping: True

- Filter out Features with Bad Peaks Only: True

- Zig-Zag Index Threshold: 0.2

- Jaggedness Threshold: 0.4

- Modality Threshold: 0.9

- Remove Potentially False Positive Isotopes: True

5. Compound Detection:

- Ions: [2M-H]-1; [M-CO2-H]-1; [M-H]-1; [M-H-H2O]-1

- Base Ions: [M-H]-1

- Remove Singlets: True

6. AcquireX Settings:

- Detect Persistent Background Ions: False

Processing node 4: Group Compounds

----

1. General Settings:

- Mass Tolerance: 2 ppm

- RT Tolerance [min]: 0.1

- Align Peaks: False

- Preferred Ions: [M-H]-1

- Area Integration: Most Common Ion

2. Peak Rating Contributions:

- Area Contribution: 3

- CV Contribution: 1

- FWHM to Base Contribution: 5

- Jaggedness Contribution: 5

- Modality Contribution: 5

- Zig-Zag Index Contribution: 5

3. Peak Rating Filter:

- Peak Rating Threshold: 3

- Number of Files: 1

Processing node 41: Fill Gaps

----

1. General Settings:

- Mass Tolerance: 2 ppm

- S/N Threshold: 1.5

- Use Real Peak Detection: True

- Apply Restrictive Gap Filling: True

Processing node 18: Mark Background Compounds

----

1. General Settings:

- Max. Sample/Blank: 5

- Max. Blank/Sample: 0

- Hide Background: True

Processing node 11: Assign Compound Annotations

----

1. General Settings:

- Mass Tolerance: 1 ppm

2. Data Sources:

- Data Source #1: Predicted Compositions

- Data Source #2: mzCloud Search

- Data Source #3: ChemSpider Search

- Data Source #4: (not specified)

- Data Source #5: (not specified)

- Data Source #6: (not specified)

- Data Source #7: (not specified)

3. Scoring Rules:

- Use mzLogic: True

- Use Spectral Distance: True

- SFit Threshold: 20

- SFit Range: 20

4. Reprocessing:

- Clear Names: False

Processing node 36: Search ChemSpider

----

1. Search Settings:

- Database(s):

    EAWAG Biocatalysis/Biodegradation Database

    Nature Chemistry

    Sigma-Aldrich

- Search Mode: By Formula or Mass

- Mass Tolerance: 2 ppm

- Max. # of results per compound: 100

- Max. # of Predicted Compositions to be searched per Compound: 3

- Result Order (for Max. # of results per compound): Order By Reference Count (DESC)

2. Predicted Composition Annotation:

- Check All Predicted Compositions: False

Processing node 38: Search mzCloud

----

1. General Settings:

- Compound Classes: All

- Precursor Mass Tolerance: 10 ppm

- FT Fragment Mass Tolerance: 10 ppm

- IT Fragment Mass Tolerance: 0.4 Da

- Library: Autoprocessed; Reference

- Post Processing: Recalibrated

- Max. # Results: 10

- Annotate Matching Fragments: False

- Search MSn Tree: False

2. DDA Search:

- Identity Search: HighChem HighRes

- Match Activation Type: True

- Match Activation Energy: Match with Tolerance

- Activation Energy Tolerance: 20

- Apply Intensity Threshold: True

- Similarity Search: None

- Match Factor Threshold: 60

3. DIA Search:

- Use DIA Scans for Search: False

- Max. Isolation Width [Da]: 500

- Match Activation Type: False

- Match Activation Energy: Any

- Activation Energy Tolerance: 100

- Apply Intensity Threshold: False

- Match Factor Threshold: 20

Processing node 5: Predict Compositions

----

1. Prediction Settings:

- Mass Tolerance: 2 ppm

- Min. Element Counts: C H

- Max. Element Counts: C90 H190 Br3 Cl4 N4 O20 P S3

- Min. RDBE: 0

- Max. RDBE: 40

- Min. H/C: 0.1

- Max. H/C: 4.5

- Max. # Candidates: 10

- Max. # Internal Candidates: 200

2. Pattern Matching:

- Intensity Tolerance [%]: 30

- Intensity Threshold [%]: 0.1

- S/N Threshold: 5

- Min. Spectral Fit [%]: 30

- Min. Pattern Cov. [%]: 90

- Use Dynamic Recalibration: True

3. Fragments Matching:

- Use Fragments Matching: True

- Mass Tolerance: 1 ppm

- S/N Threshold: 5

Processing node 34: Merge Features

----

1. Peak Consolidation:

- Mass Tolerance: 2 ppm

- RT Tolerance [min]: 0.05

Supplementary References

1. Stein, A. F. *et al.* NOAA's HYSPLIT Atmospheric Transport and Dispersion Modeling System. *Bull. Am. Meteorol. Soc.* **96**, 2059–2077 (2015).

2. Wang, Y. *et al.* The secondary formation of organosulfates under interactions between biogenic emissions and anthropogenic pollutants in summer in Beijing. *Atmos. Chem. Phys.* **18**, 10693–10713 (2018).

3. Wang, Y. *et al.* Organosulfates in atmospheric aerosols in shanghai, china: Seasonal and interannual variability, origin, and formation mechanisms. *Atmos. Chem. Phys.* **21**, 2959–2980 (2021).

4. Ma, J. *et al.* Nontarget Screening Exhibits a Seasonal Cycle of PM2.5Organic Aerosol Composition in Beijing. *Environ. Sci. Technol.* **56**, 7017–7028 (2022).

5. Glasius, M. *et al.* Chemical characteristics and sources of organosulfates, organosulfonates, and carboxylic acids in aerosols in urban Xi'an, Northwest China. *Sci. Total Environ.* **810**, 151187 (2022).

6. Stone, E. A., Yang, L., Yu, L. E. & Rupakheti, M. Characterization of organosulfates in atmospheric aerosols at Four Asian locations. *Atmos. Environ.* **47**, 323–329 (2012).

7. Le Breton, M. *et al.* Online gas- and particle-phase measurements of organosulfates, organosulfonates and nitrooxy organosulfates in Beijing utilizing a FIGAERO ToF-CIMS. *Atmos. Chem. Phys.* **18**, 10355–10371 (2018).

8. Chen, Y. *et al.* Seasonal Contribution of Isoprene-Derived Organosulfates to Total Water-Soluble Fine Particulate Organic Sulfur in the United States. *ACS Earth Sp. Chem.* **5**, 2419–2432 (2021).

9. Wang, Y. *et al.* Important Roles and Formation of Atmospheric Organosulfates in Marine Organic Aerosols: Influence of Phytoplankton Emissions and Anthropogenic Pollutants. *Environ. Sci. Technol.* **57**, 10284–10294 (2023).

10. Shakya, K. M. & Peltier, R. E. Investigating missing sources of sulfur at Fairbanks, Alaska. *Environ. Sci. Technol.* **47**, 9332–9338 (2013).

11. Dan Dan, H., Jie Li, Y., P. Lee, B. & K. Chan, C. Analysis of Organic Sulfur Compounds in Atmospheric Aerosols at the HKUST Supersite in Hong Kong Using HR-ToF-AMS. *Environ. Sci. Technol.* **49**, 3672–3679 (2015).

12. Shakya, K. M. & Peltier, R. E. Non-sulfate sulfur in fine aerosols across the United States: Insight for organosulfate prevalence. *Atmos. Environ.* **100**, 159–166 (2015).

13. P. Tolocka, M. & Turpin, B. Contribution of Organosulfur Compounds to Organic Aerosol Mass. *Environ. Sci. Technol.* **46**, 7978–7983 (2012).

14. Farmer, D. K. *et al.* Response of an aerosol mass spectrometer to organonitrates and organosulfates and implications for atmospheric chemistry. *Proc. Natl. Acad. Sci. U. S. A.* **107**, 6670–6675 (2010).

15. He, K. *et al.* The characteristics of PM2.5 in Beijing, China. *Atmos. Environ.* **35**, 4959–4970 (2001).

16. Lukács, H. *et al.* Quantitative assessment of organosulfates in size-segregated rural fine aerosol. *Atmos. Chem. Phys.* **9**, 231–238 (2009).

17. Surratt, J. D. *et al.* Organosulfate formation in biogenic secondary organic aerosol. *J. Phys. Chem. A* **112**, 8345–8378 (2008).
